# Effect of *Moringa oleifera* on inflammatory diseases: an umbrella review of 26 systematic reviews

**DOI:** 10.3389/fphar.2025.1572337

**Published:** 2025-05-19

**Authors:** Thiala Soares Josino da Silva Parente, Mariáurea Matias Sarandy, Edilane Rodrigues Dantas de Araújo, Reggiani Vilela Gonçalves, Silvana Maria Zucolotto

**Affiliations:** ^1^ Laboratory of Pharmacognosy, Department of Pharmacy, Pharmacy Faculty, Federal University of Rio Grande do Norte, Natal, Rio Grande do Norte, Brazil; ^2^ Department of General Biology, Federal University of Viçosa, Viçosa, Minas Gerais, Brazil; ^3^ Department of Animal Sciences, Plants for Human Health Institute, NC Research Campus, NC, State University, Kannapolis, NC, United States; ^4^ Animal Biology Department, Federal University of Viçosa, Viçosa, Minas Gerais, Brazil

**Keywords:** *Moringa oleifera*, inflammatory diseases, herbal medicine, systematic review, umbrella review

## Abstract

**Objectives:**

To summarize and critically assess the quality of evidence from Systematic reviews (SRs) and meta-analyses (MAs) that have evaluated the effectiveness of *Moringa oleifera* (MO) in treating inflammatory diseases and understand the main pathways activated during this exposure.

**Methods:**

A systematic search of the literature was conducted from inception until 04 November 2024, using Embase, Scopus, Web of Science, PubMed/Medline, and Cochrane Library databases. The eligibility criteria were (i) SRs on MO; (ii) SRs on MO related to inflammatory diseases; (iii) No language, year, and model limitation. Literature selection and data extraction were conducted by two independent reviewers. The quality of SRs was evaluated using the PRISMA checklist and AMSTAR-2 tool adapted.

**Results and Discussion:**

Twenty-six SRs were included, covering a total of 573 primary articles. MO leaves were the most used parts of the plant; decoction was the main extraction method; ingestion of encapsulated powder, in tablets or added with a meal were the main method of preparation; water and ethanol were the most used solvents; and flavonoids, phenolic acids and isothiocyanates were the main constituents involved in the activities of MO. Many SRs showed a promising efficacy of MO for diabetes mellitus, obesity, cancer, hypertension, dyslipidemia, among other conditions, but the quality of these SRs is questionable. Only 6 SRs indicated that they followed PRISMA (2020), and, nevertheless, they did not reach even 80% of compliance with the checklist in our evaluation. The SRs was classified, predominantly, as of low methodological quality (≤7/16) after applying AMSTAR-2. NF-kB and Nrf2 appear to be the pathways involved in the anti-inflammatory and antioxidant mechanisms of MO, respectively.

**Conclusion:**

MO is promising herbal medicine for healthcare, beneficial for inflammatory diseases, however, considering the lower level of the quality of different studies, in which the majority displayed a lack of standardization in their protocol (dose and pharmaceutical form used, use of plant powder instead of the extract, type of extraction, identification and quantification of different phytochemical markers), more well-design studies are required to confirm the conclusion.

**Systematic Review Registration:**

Registration: https://www.crd.york.ac.uk/PROSPERO, identifier CRD42022367195.

## 1 Introduction

Diseases with inflammatory etiopathology are rising in incidence worldwide (Yatoo et al., 2018). Inflammation is a serious global concern that has a detrimental impact on people’s health and wellbeing, as it causes debilitation of the patient, leading to suffering and loss of productivity, besides requiring lifelong therapy (Wylezinski et al., 2019; Khumalo et al., 2022). Chronic inflammation, caused by abnormal inflammatory activity, plays a crucial role in the development of various pathological disorders including rheumatoid arthritis, osteoarthritis, Alzheimer’s disease, cancer, obesity, and diabetes as well as cardiovascular and chronic respiratory diseases (Khumalo et al., 2022). These inflammatory diseases represent 60% of the cause of death worldwide, being considered the greatest threat to human health by the World Health Organization (WHO) and may even increase dramatically in the next 30 years (Pahwa et al., 2023). Inflammatory diseases and related conditions incur an immense social and economic burden on society. Pathologies such as multiple sclerosis (MS), ulcerative colitis (UC), Crohn’s disease (CD), and rheumatoid arthritis (RA) cost the U.S. healthcare system an aggregated total of over $35 B per year in direct costs alone. Due to their incurable progressive nature, inflammatory disorders result in a financial onus that all parties face for the remainder of a patient’s life (Wylezinski et al., 2019).

Several pharmaceutical therapies to treat inflammation are already available. The anti-inflammatory therapeutic protocols applied in this field include steroids, non-steroidal anti-inflammatory, and immunosuppressant drugs (Yatoo et al., 2018). However, these drugs are often accompanied by serious side effects, in addition, are costly and rarely available in all countries (Yatoo et al., 2018; Khumalo et al., 2022). To date, there are no medications available to cure chronic inflammatory conditions, hence, the search for safer complementary and alternative therapies is unquestionable. Herbal medicines represent a remarkable option for the treatment of several human diseases, their application as medicinal products have been expanding exponentially for their therapeutic properties and because they are cheaper, more easily available, and safer than conventional synthetic drugs (Leone et al., 2015; Nisar et al., 2018; Ahmad et al., 2019). In this context, the need for novel anti-inflammatory drugs with greater efficacy and fewer side effects has led to medicinal plants being studied as a potential source of new therapeutic agents for the treatment of inflammatory diseases (Ribeiro et al., 2018; Buabeid et al., 2022).

In this context, *M. oleifera* Lam. (Moringaceae) is a fast-growing tree native to South Asia that can be grown in any tropical and subtropical regions worldwide (Nova et al., 2020; Krawczyk et al., 2022; Silva et al., 2022), including Brazil (Rangel, 1999; Trigo et al., 2020). This plant can resist drought and mild winters, and its cultivation is possible anywhere in the world (Nova et al., 2020; Silva et al., 2022; Mthiyane et al., 2022). It is commonly known as the “tree of life”, “drumstick tree”, “horseradish tree” or “miracle tree” (Krawczyk et al., 2022; Ali Redha et al., 2021; Kashyap et al., 2022). *Moringa oleifera* (MO) is considered a very valuable plant due to all its parts can be utilized in a diet or as medicine and another industrial purpose (e.g., water purification and biofuel) since they are rich in minerals, proteins, vitamins, polyphenols, flavonoids, glucosinolates, isothiocyanates, alkaloids, tannins, and saponins (Krawczyk et al., 2022; Trigo et al., 2020; Mthiyane et al., 2022; Ali Redha et al., 2021; Kashyap et al., 2022). MO has been reported for several pharmacological activities including neuroprotective, antimicrobial, antiasthmatic, anti-malaria, cardioprotective, antidiabetic, antiobesity, hepatoprotective and anticancer (Popoola et al., 2020). Specifically, regarding anti-inflammatory and antioxidant activities, it is possible that one of the mechanisms involved is routed through the vital NF-κB and Nrf2 pathway. Studies suggest that MO can suppress the NF-κB protein and its translocation to the nucleus, which may result in the downregulation of pro-inflammatory genes. Besides that, MO may be able to upregulate Nrf2, leading to increased transcription of antioxidants and cytoprotective genes as well as anti-inflammatory cytokines (Kashyap et al., 2022; Popoola et al., 2020). Among these activities, the effect of attenuating the negative impact of chronic inflammation and acting against its associated disorders has been highly evidenced (Mthiyane et al., 2022; Xiao et al., 2020). Such anti-inflammatory capability has been attributed to the glucosinolates, flavonoids, and phenolic acids content in the MO. All these compounds allow the MO to be explored as a cheap and effective drug source to treat inflammation-related diseases (Nova et al., 2020; Popoola et al., 2020).

From the year 2000 up to 2020, 2,345 articles associated with MO have been published on the Scopus database. In the last years, 2016–2020, research outputs increased by 50% (George et al., 2021). A review in the literature indicates much preclinical evidence on animal models that support the pharmacological properties and safety of MO. However, few reports about safety based on clinical trials have been published. Tied to this, there are present methodological limitations in these studies–non-standardization of dose and pharmaceutical form, use of dried plant powder instead of extract, lack of identification and quantification of markers, among others–which diminishes the scientific evidence of findings. Among these publications, several systematic reviews (SRs) or meta-analyses (MAs) have investigated the use of MO for treating certain conditions, but its results are not consistent, and the methodological quality of some SRs is unknown. In addition, to date, an overview was not found that focused on the efficacy and safety of MO to treat inflammatory conditions. Thus, the purpose of this study was to summarize and critically assess the quality of evidence from SRs and MAs that have evaluated the effectiveness of MO in treating inflammatory diseases and understand the main pathways activated during this exposure.

## 2 Materials and methods

**FIGURE 1:**
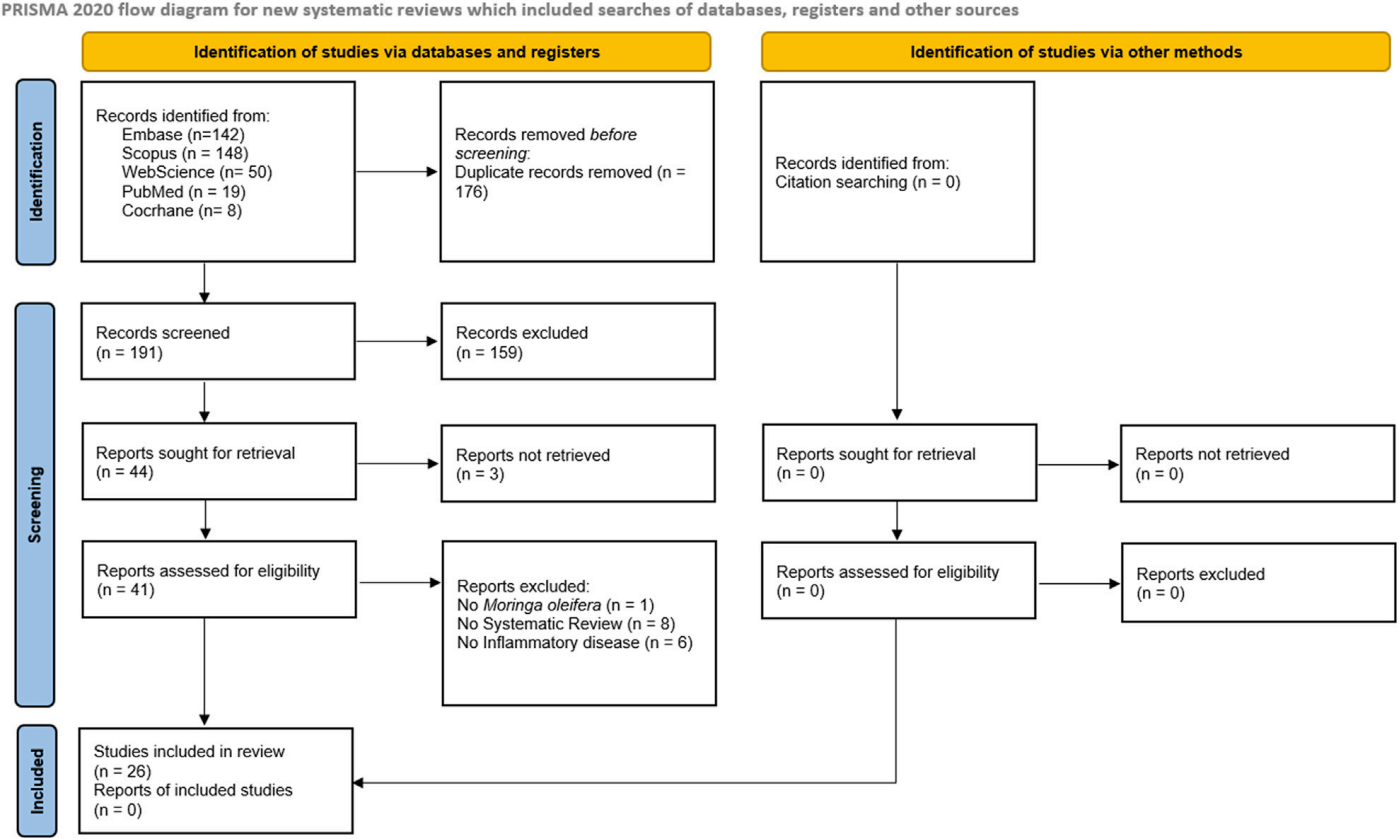
Preferred Reporting Items for Systematic Reviews and Meta-Analyses (PRISMA) flow diagram. The flowchart indicates the research records obtained at all standardized stages of the search process required for the development of systematic reviews and meta-analyses. Based on the PRISMA statement (http://www.prisma-statement.org, accessed on 24 October 2022).

This review was conducted in adherence with the Preferred Reporting Items for Systematic Reviews and Meta-Analyses (PRISMA) 2020 guidelines (Page et al., 2021) (Figure 1). The methods were adapted from the Cochrane protocol for overviews of reviews (Pollock et al., 2023).

### 2.1 Research question

The main question to be answered in this systematic review was: What is the effect of *M. oleifera* (MO) on inflammatory diseases using systematically reviewed pre-clinical and clinical studies? What are the main inflammatory pathways controlled after the MO exposure?

The protocol was registered on 19 November 2022 (https://www.crd.york.ac.uk/PROSPERO; registration number: CRD42022367195). The GRADE system (the Grading of Recommendations Assessment Development and Evaluation) was not applied.

### 2.2 Search strategy and study selection

Based on two parameters, a search strategy was developed to maximize the retrieval of relevant study registers. The first parameter was based on a direct advanced search in the electronic databases Embase, Scopus, Web of Science, PubMed/Medline, and Cochrane Library (all accessed on 04 November 2024). In the second parameter, indirect screening was performed by carefully reading the reference list of selected articles previously in the databases, to select potential studies to be included in the systematic review. For all databases, search filters were based on: (i) *M. oleifera* and (ii) Systematic Review and Meta-analysis.

A search filter was initially developed for PubMed/Medline according to standardized descriptors (MeSH terms) organized in the hierarchical tree of the MeSH database. The commands (MeSH Terms) and TIAB were combined to broaden the retrieval of relevant indexed studies and those in the indexing process. Supplementary Data S1 fully describes the search strategy for this review.

Two reviewers (TSJSP and MMS) conducted the literature search, removed duplicate articles, and examined titles and abstracts for eligibility criteria. After initial screening, full-text articles from potentially relevant studies were independently certified for eligibility by two reviewers (TSJSP and MMS). The kappa test was performed for the selection of articles (kappa = 0.869). Selections were then compared, and inconsistencies were resolved in consultation with two other reviewers (RVG and SMZL).

### 2.3 Eligibility criteria

Only studies that met the following eligibility criteria were selected: (i) Systematic reviews on *M. oleifera*; (ii) Systematic reviews on *M. oleifera* related to inflammatory diseases; (iii) No language, year, and model limitation. Primary studies, narrative reviews, scoping reviews, systematic reviews considered the association of MO with other plants and systematic reviews related to other diseases that did not involve inflammation were excluded. The detailed criteria following “PICOS” principle (P: Participants, I: Intervention, C: Control, O: Outcomes, S: Study designs) were shown in Table 1.

**TABLE 1:** The inclusion criteria following “PICOS” principle.

Category	Inclusion criteria
Population	Cells, animal, and human without any restriction
Intervention	*Moringa oleifera* (any dosage, preparation, and duration)
Comparator	Placebo or active drug or treatment as usual another
Outcome	Measure(s) (inflammatory diseases)
Study design	Systematic review and meta-analysis

### 2.4 Data extraction

Two independent reviewers (TSJSP and MMS) will conduct the data extraction blindly. Then, data will be compared between reviewers, and conflict information will be resolved by two more reviewers (RVG and SMZL). The essential data from each study were extracted through structured tables according to the following descriptive levels: (i) Characteristics of systematic reviews (first author, year and study location number of included studies, search databases, search time, registration number, quality assessment methods for included studies, meta-analysis or not, *etc.*); (ii) Characteristics of the experimental model (species, sex, age, weight); (iii) Information about *Moringa oleifera* treatment (plant part used, type of extract, dose, frequency of administration, and pharmaceutical form); (iv) Primary outcomes (mechanisms involved in inflammatory and oxidative processes); (v) Secondary outcomes (associated pathologies). The word cloud was elaborated using a free online word cloud generator (www.wordclouds.com). A study location map was elaborated using a free interactive map generator (www.mapinseconds.com). All other data was processed using Microsoft Office Excel^®^ software.

### 2.5 Quality assessment

Initially, to assess adherence to the PRISMA tool, all the studies were submitted to this checklist in its latest version (2020). The items were classified as meets (✓) or do not meet (x). Each SR was scored based on compliance with the criteria, through the proportion of criteria met *versus* total criteria. The average percentage of adherence of the 26 SRs to the PRISMA tool was also calculated. Fourteen of the 42 PRISMA items and sub-items (12, 13a-13f, 14, 19, 20a-20d and 21) are specific to MAs, and were outlined as “NA” if the SR did not contain an MA. Thus, all SRs that did not lead to an MA were scored on 28 items instead of 42. The most adhered-to criterion among the SRs was also measured. The score was calculated as the proportion of SRs that met the criterion *versus* the total number of SRs in which the criterion was applied.

Subsequently, the articles containing *in vivo* studies (animal and human) were assessed using the modified AMSTAR-2 instrument. AMSTAR-2 is a 16-item checklist critical appraisal instrument for SRs containing randomized clinical trials, non-randomized clinical trials or both studies on health interventions. As the AMSTAR-2 was developed for synthesizing SRs from controlled clinical trials, we adapted it from Rocque et al. (2021), to suit a research context in which most of the SRs included did not include these types of trials, thus also being able to assess the quality of non-clinical (*in vivo*) studies. Each SR was scored based on whether the criteria were met (“yes” = 1 point), partially met (“partial yes” = 0.5 point) or not met (“no” = 0 point) for each of the 16 items. Three of the 16 AMSTAR-2 items (Krawczyk et al., 2022; Silva et al., 2022; Mthiyane et al., 2022) are specific to SRs containing MAs and were therefore delineated as “NA,” thus not scoring if the SR did not contain an MA. The scores were then calculated as a proportion of criteria met, based on Yuan et al. (2017). The AMSTAR-2 calculator assigned each study a final critical assessment of methodological quality of “high” (≥12/16) or “low” (≤7/16). The full modifications can be found in Supplementary Data S2.

An additional analysis was carried out to weigh up the relationship between the impact factor of the journals in which the SRs were published, the year of publication, the PRISMA checklist used (2009 or 2020) and the use of methodological quality assessment tools for individual studies included in each SR. The assessment of which version of PRISMA was used by each SR was made by analyzing the bibliographic reference cited by the SR in its methodology and/or the diagram of the study selection procedure, since there is a difference between the two versions (PRISMA 2009 or 2020).

## 3 Results

### 3.1 Study selection and characteristics of included studies

As shown in the PRISMA diagram in Figure 1, we have obtained an initial set of 367 references. A total of 176 studies were duplicated, and 159 with inadequate themes were excluded after reading the title and abstract. Of the 44 remaining studies, 3 studies were not retrieved in full, and 15 articles were excluded after reading the full text for not meeting the eligibility criteria. The reference list of all included studies was analyzed to ensure the identification of additional relevant studies, but none were included. Therefore, 26 studies were included in the systematic review, with six articles from *in vivo* studies, four from *in vitro* studies, and eleven from both, two from *in vivo*, *in vitro* and *in silico*, plus one *in silico*, one ethnomedicinal, and one ethnobotanical. The flowchart and each step performed in the selection process to retrieve relevant studies are shown in Figure 1.

**FIGURE 2:**
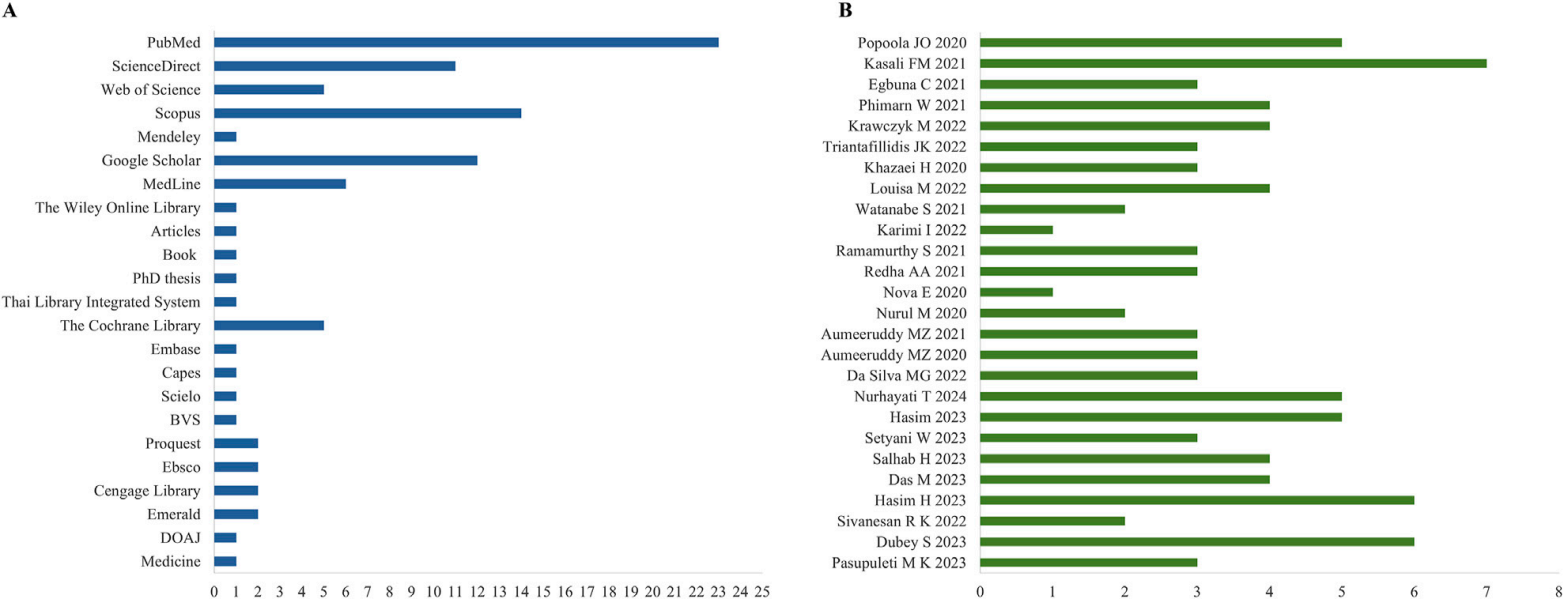
**(A)** Databases used in SRs to search for primary articles. The *x*-axis represents the number of reviews per database; **(B)** The *x*-axis represents the number of databases used in each review.

Most authors used long time intervals of research in the eligibility criteria, specifying start and end times (years): 49 (1970–2019) (Aumeeruddy and Mahomoodally, 2020), 32 (1988–2020) (Aumeeruddy and Mahomoodally, 2021), 29 (1990–2019) (Popoola et al., 2020), and 28 years (1993–2021) (Triantafillidis et al., 2022); while some did not specify the date of entry into the database, stating that the research period began from inception, e.g., inception to December 2019 (Phimarn et al., 2021), and inception to first of August 2021 (Louisa et al., 2022). One paper had a short search variation, less than a year: August 2019 to April 2020 (Nova et al., 2020), and one did not report the research period (Sivanesan et al., 2022). The most searched databases were PubMed, Scopus, Google Scholar, and Web of Science (Figure 2A) whereas the study with the highest number of databases consulted was Kasali et al. (2021) (n = 7) (Figure 2B). Among the 26 SRs analyzed, 521 primary articles were included. A median of 9.5 primary studies were included in each systematic review (mean = 20.04; SD = 25.87; range 1–109). Of the 26 reviews, 7 SRs (26.92%) performed MAs, and the remaining SRs (73.08%) did not (Supplementary Data S3).

**FIGURE 3:**
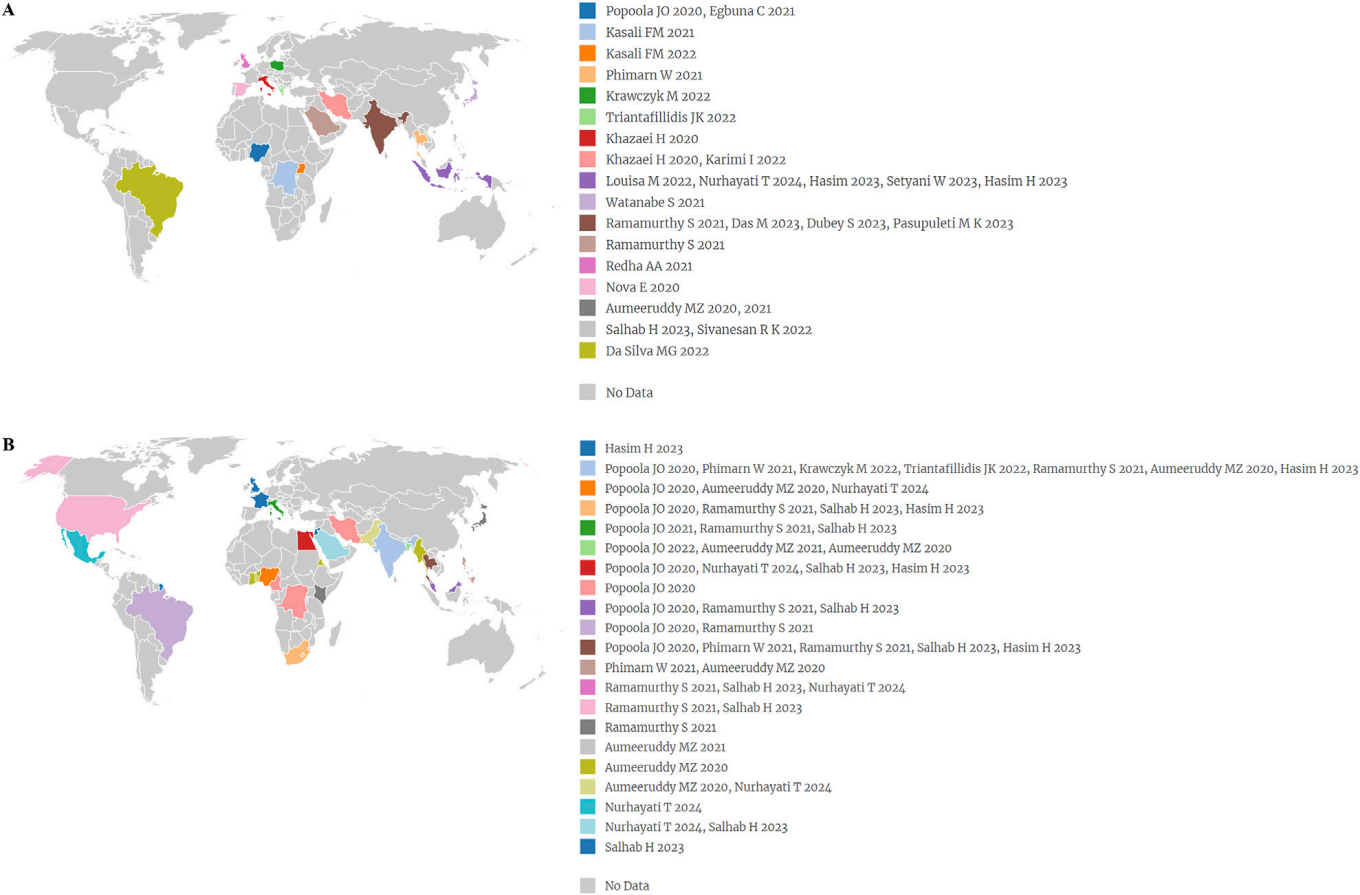
Distribution of the included articles by country. **(A)** According to first author and corresponding author; **(B)** According to the origin of the primary articles.

The included systematic reviews’ authors were concentrated in four continents Asia, Africa, Europe and South America (Figure 3A). Countries of origin by continents include (from highest to lowest citation frequency, then by alphabetical order): Asia (Kashyap et al., 2022): Bahrain (Yatoo et al., 2018), India (Pahwa et al., 2023), Indonesia (Leone et al., 2015), Iran (Wylezinski et al., 2019), Japan (Yatoo et al., 2018), Saudi Arabia (Yatoo et al., 2018), Thailand (Yatoo et al., 2018), Vietnam (Wylezinski et al., 2019); Africa (Nisar et al., 2018): Democratic Republic of Congo (Yatoo et al., 2018), Mauritius (Wylezinski et al., 2019), Nigeria (Wylezinski et al., 2019), Uganda (Yatoo et al., 2018); Europe (Leone et al., 2015): Italy (Yatoo et al., 2018), Greece (Yatoo et al., 2018), Poland (Yatoo et al., 2018), Spain (Yatoo et al., 2018), United Kingdom (Yatoo et al., 2018); South America (Yatoo et al., 2018): Brazil (Yatoo et al., 2018). Regarding the location of the primary articles, half of the SR did not report this data (n = 14; 53.85%) and the other half targeted mainly Asia (n = 11) and Africa (n = 9), but also America (n = 5) and Europe (n = 4) (Figure 3B). India ranked first (n = 7), followed by Thailand (n = 5), Egypt (n = 4), South Africa (n = 4), Bangladesh (n = 3), Malaysia (n = 3), Korea (n = 3), Nigeria (n = 3), Italy (n = 3), Philippines (n = 2), Pakistan (n = 2), Brazil (n = 2), Arabia (n = 2), United States (n = 2), Iran (n = 1), Japan (n = 1), Myanmar (n = 1), Cameroon (n = 1), Democratic Republic of the Congo (n = 1), Kenya (n = 1), Mexico (n = 2), Burkina Faso (n = 1), Benin (n = 1), Eritreia (n = 1), Ghana (n = 1), Mauritius (n = 1), Myanmar (n = 1), Reuniun (n = 1), Rodrigues (n = 1), Sierra (n = 1), Togo (n = 1), Saudi Jordan (n = 1), Indochina (n = 1), France (n = 1) and United Kingdom (n = 1).

**FIGURE 4:**
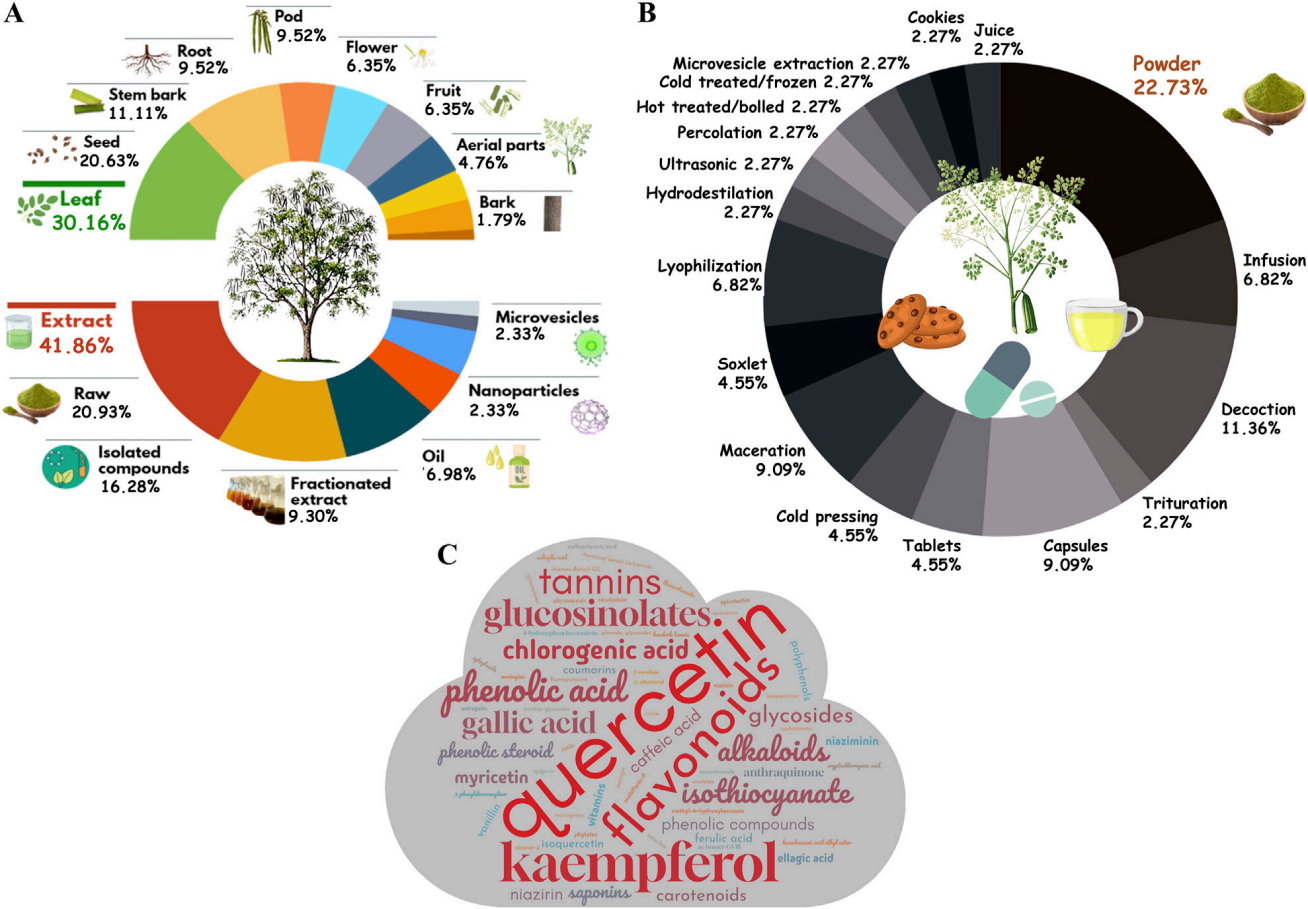
**(A)** Percentage of the different parts and forms of MO used; **(B)** Percentage of use of various methods of extraction/preparation; **(C)** Word clouds elaborated from the phytochemicals cited in the included SRs. The size of each word is proportional to the number of systematic reviews in which the compound appears.

Analysis of the plant parts revealed that leaf (n = 19) was mostly used, followed by seed (n = 13), stem bark (n = 7), pod (n = 6), root (n = 6), flower (n = 4), fruit (n = 4), aerial parts (n = 3), and bark (n = 1). Regarding the forms used, the most preferred was the extract (n = 18), followed by raw (n = 9), isolated compounds (n = 7), fractionated extract (n = 4), oil (n = 3), nanoparticles and microvesicles (n = 1, each) (Figure 4A). The MO part used was not reported in six SRs (Krawczyk et al., 2022; Triantafillidis et al., 2022; Egbuna et al., 2021; Hasim et al., 2023a; Hasim et al., 2023b; Pasupuleti et al., 2023), while the form used was also not reported in seven (Triantafillidis et al., 2022; Egbuna et al., 2021; Khazaei et al., 2021; Hasim et al., 2023a; Hasim et al., 2023b; Watanabe et al., 2021; Da Silva et al., 2022).

The present review revealed that powder (n = 10), decoction (n = 5), capsules (n = 4) and maceration (n = 4), were the most often used methods of extraction/preparation. Other less reported were infusion (n = 3), lyophilization (n = 3), cold pressing (n = 2), soxhlet (n = 2), tablets (n = 2), and other methods were described in one paper each (n = 5) (Figure 4B). The methods of extraction/preparation were not reported by some authors (n = 14) (Krawczyk et al., 2022; Triantafillidis et al., 2022; Egbuna et al., 2021; Khazaei et al., 2021; Hasim et al., 2023a; Hasim et al., 2023b; Pasupuleti et al., 2023; Watanabe et al., 2021; Da Silva et al., 2022; Nurul and Harun, 2020; Salhab et al., 2023; Das et al., 2023; Sivanesan et al., 2022; Dubey et al., 2023). It is important to mention that in the 19 clinical trials analyzed within the SRs included in this overview, there is a predominance of the use of MO leaf. When carrying out this analysis, it was observed that Ali Redha et al. (2021) mistakenly classified the type of extract used in the clinical trial by Ezzat et al. (2020), as they reported that the extract used had been MO leaves petroleum ether extract instead of MO leaves 70% ethanol extract powder (Supplementary Data S4).

As shown in Supplementary Data S3B, solvents commonly used in the extraction of MO are polar (e.g., water, ethanol, methanol), intermediate polar (e.g., dichloromethane), and nonpolar (e.g., n-hexane, ethyl acetate). Among the solvents that have been utilized in the preparation of extracts of MO, water was more commonly used (22.80%), ethanol (21.05%), and methanol (19.30%), and other solvents were used in 40.36% of the studies. Eleven studies did not report such information, which represents 42.30% of the SRs included (Krawczyk et al., 2022; Aumeeruddy and Mahomoodally, 2021; Triantafillidis et al., 2022; Phimarn et al., 2021; Kasali et al., 2021; Egbuna et al., 2021; Khazaei et al., 2021; Pasupuleti et al., 2023; Watanabe et al., 2021; Da Silva et al., 2022; Das et al., 2023).

For the analysis of the effective chemical composition, word clouds were elaborated from the main phytochemicals cited in each included SR to report the main classes and secondary metabolites responsible for the therapeutic actions of MO (Figure 4C). Therefore, the number of times (n) that each word appears means the number of SRs that quoted it. For the phytochemical classes, the words flavonoids (n = 14), alkaloids (n = 8), glucosinolates (n = 7), glycosides (n = 6), tannins (n = 7), phenolic acids (n = 6) were most frequently quoted. Similarly, the words are among the most cited quercetin (n = 15), kaempferol (n = 9), chlorogenic acid (n = 7), gallic acid (n = 6), isoquercetin (n = 5), moringin (n = 4) in terms of the most relevant secondary metabolites. Of the 26 SRs, 6 studies (23.07%) did not report any information about the chemical composition of MO (Aumeeruddy and Mahomoodally, 2020; Aumeeruddy and Mahomoodally, 2021; Triantafillidis et al., 2022; Pasupuleti et al., 2023; Sivanesan et al., 2022; Dubey et al., 2023).

**FIGURE 5:**
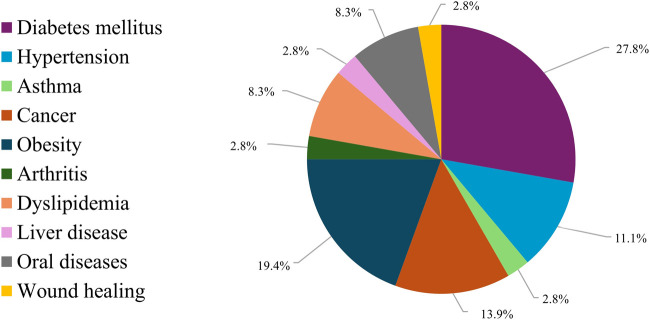
Citation frequency of the main inflammatory diseases reported in the included SRs.

Figure 5 presents the citation frequencies of the most reported inflammatory diseases in the included SRs, with diabetes mellitus showing the highest number (n = 10; 27.8%).

The analysis of the experimental design was also carried out. Eight studies (30.76%) reported the MO doses used, which ranged from 40 mg to 6,400 mg of extracts in animal studies (Nova et al., 2020; Ali Redha et al., 2021; Popoola et al., 2020; Louisa et al., 2022; Salhab et al., 2023; Das et al., 2023; Nurhayati et al., 2024; Setyani et al., 2023), from 0.03 g/kg to 50 g of powder and 400 mg of extract in human studies (Nova et al., 2020; Ali Redha et al., 2021; Phimarn et al., 2021; Louisa et al., 2022; Das et al., 2023; Sivanesan et al., 2022) and 10 ng/mL to 1 mg/mL of extracts in *in vitro* studies (Louisa et al., 2022; Salhab et al., 2023). Ten SRs (38.46%) reported duration of treatments, which ranged from 1 day to 24 weeks in animal studies (Nova et al., 2020; Popoola et al., 2020; Louisa et al., 2022; Watanabe et al., 2021; Salhab et al., 2023; Das et al., 2023; Sivanesan et al., 2022; Setyani et al., 2023) and from 1 day to 90 days in human studies (Nova et al., 2020; Ali Redha et al., 2021; Phimarn et al., 2021; Louisa et al., 2022; Das et al., 2023; Sivanesan et al., 2022). The most used route of administration was the oral route for both humans (pharmaceutical form or addition to the meal) and animals (diet or gavage), with eleven SRs (42.3%) reporting this information (Nova et al., 2020; Ali Redha et al., 2021; Popoola et al., 2020; Louisa et al., 2022; Khazaei et al., 2021; Watanabe et al., 2021; Nurul and Harun, 2020; Salhab et al., 2023; Das et al., 2023; Sivanesan et al., 2022; Setyani et al., 2023). Finally, thirteen reviews (Nova et al., 2020; Krawczyk et al., 2022; Ali Redha et al., 2021; Popoola et al., 2020; Phimarn et al., 2021; Louisa et al., 2022; Watanabe et al., 2021; Salhab et al., 2023; Sivanesan et al., 2022; Nurhayati et al., 2024; Setyani et al., 2023; Ramamurthy et al., 2021; Karimi, 2022) reported some information about the evaluated population (e.g., species, sex, age, number of participants or weight), with the articles by Watanabe et al. (2021), Nova et al. (2020), Setyani et al. (2023), Salhab et al. (2023) and Sivanesan et al. (2022) being the more complete.

The adverse events using MO as an intervention were scarcely reported, only in 5 SRs (Nova et al., 2020; Popoola et al., 2020; Phimarn et al., 2021; Louisa et al., 2022; Nurhayati et al., 2024). Apparently, MO appears to be safe, showing some adverse events only at high doses. Details about the design of the experiments can be found in Supplementary Data S3.

### 3.2 Markers and mediators involved in *Moringa oleifera* activities

**FIGURE 6:**
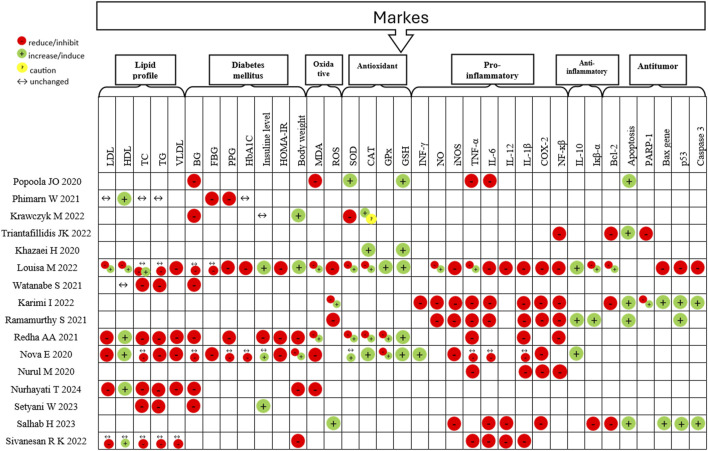
Results of the outcomes of each analyzed. 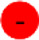 reduce/inhibit; 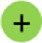, increase/induce; 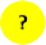, caution; ↔, unchanged. LDL, low-density lipoprotein; HDL, high-density lipoprotein; TC, total cholesterol; TG, triglyceride; VLDL, very low-density lipoprotein; BG, blood glucose; FBG, fasting blood glucose; PPG, postprandial glucose; HbA1C, Hemoglobin A1c; HOMA-IR, Homeostasis Model Assessment- Insulin Resistance; MDA, malondialdehyde; ROS, reactive oxygen species; SOD, superoxide dismutase; CAT, catalase; GPx, glutathione peroxidase; GSH, glutathione; INF- γ interferon-gamma; NO, nitric oxide; iNOS, inducible nitric oxide synthase; TNF-α, tumor necrosis factor alpha; IL-6, interleukin 6; IL-12, interleukin 12; IL-1β, interleukin 1β; COX-2, cyclooxygenase-2; NF-κβ, nuclear factor- κβ; IL-10, interleukin 10; Iκβ-α, inhibitor of κβ; Bcl2, B-cell lymphoma 2; PARP-1, Poly(ADP-ribose) polymerase-1.

The analysis of the markers and mediators involved in MO activities was performed in 61.54% of the studies (n = 16). The most cited outcomes in these 12 SRs were classified into lipid profile (30.77%, n = 8), diabetes mellitus (34.62%, n = 9), oxidative stress (38.50%, n = 10), inflammation (38.50%, n = 10), and antitumor (23.07%, n = 6) and results are shown in Figure 6.

#### 3.2.1 *M. oleifera* on lipid profile markers

The analyzed lipid profile markers were low-density lipoprotein (LDL) (23.07%, n = 6), high-density lipoprotein (HDL), triglyceride (TG) and total cholesterol (TC) (26.92%, n = 7 in each), in addition to very low-density lipoprotein (VLDL) (19.23%, n = 5). The results of HDL, TC and TG varied between clinical trials (Phimarn et al., 2021; Sivanesan et al., 2022) and animal experiments (Hasim et al., 2023a; Sivanesan et al., 2022). The results of the LDL, HDL, TC, and TG varied within the same experimental model: increase and decrease in LDL levels in humans, while in animals only reduction; increase and decrease in HDL levels in animals, while only increases in humans; increase and reduction of TC levels in humans, while reduction and no change in levels in animals; decrease and non-change in TG levels in animals. TG levels in humans have only been assessed by Phimarn et al. (2021) and Sivanesan et al. (2022). VLDL levels were reduced in all tests performed, but they were only evaluated in animal models. Despite the variation between the presented results, a predominance of reduction in LDL, TG, TC, and VLDL levels and an increase in HDL levels were observed (Figure 6).

#### 3.2.2 *M. oleifera* on diabetes mellitus markers

The most evaluated outcomes among the included SRs were blood glucose (BG) (30.76%, n = 8), fasting plasma glucose (FBS), hemoglobin A1c (HbA1C), and homeostasis model assessment-insulin resistance (HOMA-IR) (11.53%, n = 3 in each), besides postprandial glucose (PPG) (15.38%, n = 4), insulin level (19.23%, n = 5), and body weight (23.07%, n = 6). However, there were some inconsistencies in the results due to the different responses obtained between animal and human studies. The results indicated that MO had a positive effect, reduction of levels, for controlling BG, PPG, and FBG in both animals and humans, however Nova et al. (2020) presented human studies in which there was no difference in plasma glucose (n = 2) and PPG levels (n = 1) between the treated and control groups, as well as Louisa et al. (2022) also reported no change in plasma glucose levels (n = 2) and FBG (n = 1) in human studies. Three reviews investigated the effect of MO on HbA1C levels, the authors found that HbA1C can be reduced in animal and human models, however, Phimarn et al. (2021) and Nova et al. (2020) reported there were no changes in human studies included in their reviews. Insulin was another biomarker with inconsistent results, since SRs demonstrated its increase, decrease, and unchanged in animal studies. Finally, the action of MO on body weight was also inconsistent, as both an increase and a decrease in this outcome were observed in animal models (Figure 6).

#### 3.2.3 *M. oleifera* on oxidative stress markers

The level of antioxidant markers such as superoxide dismutase (SOD), catalase (CAT), and glutathione (GSH) (19.23%, n = 5 in each), or glutathione peroxidase (GPx) (11.53%, n = 3) were analyzed in 23.07% of the studies (n = 6). While oxidative markers such as malondialdehyde (MDA) (19.23%, n = 5) or reactive oxygen species (ROS) (15.38%, n = 4) were analyzed in 30.77% of the studies (n = 8). There were some inconsistencies in the results. First, Krawczyk et al. (2022) explain in their meta-analysis that the MO extracts reduced the activity of SOD in the treated rats, lowering the oxidative stress by decreasing free-radical concentration and that the results of the CAT activity analysis should be interpreted with caution. Second, the results of the MDA, ROS, SOD, CAT and GPx varied within the same experimental model or when compared between humans and animals: increase (Ali Redha et al., 2021; Louisa et al., 2022) and decrease (Nova et al., 2020; Ali Redha et al., 2021; Popoola et al., 2020; Louisa et al., 2022; Nurhayati et al., 2024) in MDA in animals, while in humans only decrease (Ali Redha et al., 2021; Popoola et al., 2020); increase (Karimi, 2022) and decrease (Louisa et al., 2022; Ramamurthy et al., 2021; Karimi, 2022) in ROS levels in cells (increase when dealing with cancer cells), while only reduction in animals (Louisa et al., 2022); increase, reduction or no change in SOD in animals (Nova et al., 2020; Krawczyk et al., 2022; Ali Redha et al., 2021; Popoola et al., 2020; Louisa et al., 2022), while only increase in humans (Ali Redha et al., 2021); increase and decrease of CAT in animals (Nova et al., 2020; Krawczyk et al., 2022; Ali Redha et al., 2021; Louisa et al., 2022; Khazaei et al., 2021); increase and reduction of GPx in animals (Nova et al., 2020; Ali Redha et al., 2021; Louisa et al., 2022) while only increase in humans (Ali Redha et al., 2021). GSH levels were reduced in all tests performed (animal models) (Popoola et al., 2020; Louisa et al., 2022; Khazaei et al., 2021). ROS, CAT, and GSH have not been analyzed in humans. Besides, MDA, SOD, and GPx were analyzed in humans only in Nova et al. (2020). Despite the variation between the presented results, trends pointed towards a reduction in levels of oxidant markers and an increase in levels of antioxidant molecules (Figure 6).

#### 3.2.4 *M. oleifera* on inflammatory mediators

Of the main inflammatory mediators, the pro-inflammatory evaluated were interferon-gamma (INF- γ) (7.69%, n = 2), nitric oxide (NO) (11.53%, n = 3), inducible nitric oxide synthase (iNOS) (19.23%, n = 5), tumor necrosis factor-alpha (TNF-α) (30.76%, n = 8), interleukin 6 (IL-6) and interleukin 1β (IL-1β) (26.92%, n = 7 in each), cyclooxygenase-2 (COX-2) and Nuclear factor- Kβ (NF-κβ) (23.07%, n = in each), in addition, interleukin 12 (IL-12) (11.53%, n = 3). While the anti-inflammatory mediators evaluated were interleukin 10 (IL-10) and Iκβ-α (11.53%, n = 3 in each). Inflammatory and anti-inflammatory mediators have not been evaluated in clinical trials, they have only been analyzed in cells and animals. The results showed some inconsistencies, but MO’s effect tended to downregulate pro-inflammatory and upregulate anti-inflammatory mediators (Figure 6).

#### 3.2.5 *M. oleifera* on tumor markers

The level of pro-apoptotic markers such as apoptosis (19.23%, n = 5), p53 (15.38%, n = 4), caspase-3 and Bax gene (11.53%, n = 3 in each) or PARP-1 (7.69%, n = 2) were analyzed in the six studies. The antiapoptotic marker Bcl2 was analyzed in 15.38% (n = 4) of the studies. The results demonstrated an increase in the expression of the anti-apoptotic marker Bcl2 and a reduction in the expression of pro-apoptotic markers in studies with healthy cells. On the other hand, suppression of Bcl2 expression and activation of pro-apoptotic markers were observed in studies with cancer cells (Figure 6).

### 3.3 Quality assessment of the included reviews

Methodological quality for each of the included SRs is presented in Table 2 (PRISMA) and Table 3 (AMSTAR-2). It should be noted that the quality of each SR reflects the rigor and transparency of the research team rather than the quality of evidence for the MO intervention.

**TABLE 2:** Assessment of adherence to the PRISMA 2020 statement.

Section and topic	Item #	Popoola 2020	Kasali 2021	Egbuna 2021	Phimarn 2021	Krawczyk 2022	Triantafillidis 2022	Khazaei 2020	Louisa 2022	Watanabe 2021	Karimi 2022	Ramamurthy 2021	Ali Redha 2021	Nova et al., 2020	Nurul 2020	Aumeeruddy 2021	Aumeeruddy 2020	Da Silva 2022	Nurhayati 2024	Hasim 2023a	Setyani 2023	Salhab 2023	Das 2023	Hasim 2023b	Sivanesan 2022	Dubey 2023	Pasupuleti M 2023	Number of reported articles (n/n, %)
Title	1	✓	x	✓	x	✓	✓	✓	✓	✓	✓	x	✓	✓	✓	✓	✓	✓	✓	✓	x	✓	✓	✓	✓	✓	✓	22/26 (84.6)
Abstract	2	x	x	x	x	x	x	x	x	x	x	x	x	x	x	x	x	x	x	x	x	x	x	x	x	x	x	0/26 (0)
Introduction																												
Rationale	3	✓	✓	✓	✓	✓	✓	✓	✓	✓	✓	✓	✓	✓	✓	✓	✓	✓	✓	✓	✓	✓	✓	✓	✓	✓	✓	26/26 (100.0)
Objectives	4	✓	✓	x	✓	✓	✓	x	✓	✓	✓	✓	✓	✓	✓	✓	✓	✓	✓	✓	✓	✓	✓	✓	✓	✓	✓	24/26 (92.3)
Methods																												
Eligibility criteria	5	✓	x	✓	✓	x	x	x	✓	✓	✓	✓	✓	✓	x	✓	✓	✓	✓	x	x	✓	✓	x	✓	x	✓	17/26 (65.4)
Information sources	6	x	✓	✓	✓	x	✓	✓	✓	✓	✓	x	✓	✓	x	x	x	✓	x	x	✓	x	x	✓	x	✓	✓	15/26 (57.7)
Search strategy	7	✓	x	✓	✓	✓	✓	✓	✓	✓	✓	✓	✓	✓	✓	✓	✓	✓	✓	✓	x	✓	✓	✓	✓	x	✓	23/26 (88.5)
Selection process	8	✓	x	✓	✓	✓	x	✓	x	x	x	x	x	x	x	x	x	✓	✓	x	x	✓	x	✓	x	x	x	9/26 (34.6)
Data collection process	9	✓	x	✓	x	✓	x	✓	✓	✓	x	✓	x	x	x	x	x	x	✓	x	x	x	x	✓	✓	x	x	10/26 (38.4)
Data items	10a	✓	✓	✓	✓	✓	x	✓	✓	✓	x	✓	x	✓	x	x	✓	✓	✓	✓	✓	✓	x	✓	✓	✓	x	19/26 (73.1)
	10b	✓	✓	x	✓	✓	x	✓	✓	✓	✓	x	x	✓	x	x	✓	x	✓	x	✓	✓	✓	x	✓	x	✓	16/26 (61.5)
Study risk of bias assessment	11	✓	✓	x	✓	x	x	x	x	✓	x	✓	x	x	x	x	x	x	✓	x	x	x	x	x	x	x	✓	7/26 (26.9)
Effect measures	12	NA	NA	NA	✓	✓	NA	NA	NA	✓	NA	NA	NA	NA	NA	NA	NA	NA	NA	✓	NA	NA	✓	✓	NA	✓	NA	7/7 (100)
Synthesis methods	13a	NA	NA	NA	✓	✓	NA	NA	NA	✓	NA	NA	NA	NA	NA	NA	NA	NA	NA	✓	NA	NA	✓	✓	NA	x	NA	6/7 (85.7)
	13b	NA	NA	NA	✓	✓	NA	NA	NA	✓	NA	NA	NA	NA	NA	NA	NA	NA	NA	✓	NA	NA	✓	x	NA	x	NA	5/7 (71.4)
	13c	NA	NA	NA	x	✓	NA	NA	NA	x	NA	NA	NA	NA	NA	NA	NA	NA	NA	✓	NA	NA	✓	✓	NA	✓	NA	5/7 (71.4)
	13d	NA	NA	NA	✓	✓	NA	NA	NA	✓	NA	NA	NA	NA	NA	NA	NA	NA	NA	✓	NA	NA	✓	✓	NA	✓	NA	7/7 (100.0)
	13e	NA	NA	NA	✓	x	NA	NA	NA	✓	NA	NA	NA	NA	NA	NA	NA	NA	NA	✓	NA	NA	x	✓	NA	✓	NA	5/7 (71.4)
	13f	NA	NA	NA	✓	x	NA	NA	NA	✓	NA	NA	NA	NA	NA	NA	NA	NA	NA	x	NA	NA	x	x	NA	x	NA	2/7 (28.6)
Reporting bias assessment	14	NA	NA	NA	✓	x	NA	NA	NA	✓	NA	NA	NA	NA	NA	NA	NA	NA	NA	x	NA	NA	x	x	NA	x	NA	2/7 (28.6)
Certainty assessment	15	x	x	x	x	x	x	x	x	x	x	x	x	x	x	x	x	x	x	x	x	x	x	x	x	x	x	0/26 (0.0)
Results																												
Study selection	16a	✓	✓	✓	✓	✓	x	✓	✓	✓	✓	✓	✓	✓	✓	✓	✓	✓	✓	✓	✓	✓	✓	✓	✓	✓	✓	25/26 (96.2)
	16b	x	✓	✓	✓	✓	x	✓	✓	✓	✓	x	✓	✓	x	✓	✓	x	✓	✓	✓	x	✓	x	✓	x	✓	18/26 (69.3)
Study characteristics	17	✓	✓	✓	✓	✓	✓	✓	✓	✓	✓	✓	✓	✓	✓	✓	✓	✓	✓	✓	✓	✓	✓	✓	✓	✓	✓	26/26 (100.0)
Risk of bias in studies	18	✓	✓	x	✓	x	x	x	x	✓	x	✓	x	x	x	x	x	x	✓	x	x	x	x	x	x	✓	✓	8/26 (30.8)
Results of individual studies	19	NA	NA	NA	✓	✓	NA	NA	NA	✓	NA	NA	NA	NA	NA	NA	NA	NA	NA	✓	NA	NA	✓	✓	NA	✓	NA	7/7 (100.0)
Results of syntheses	20a	NA	NA	NA	x	x	NA	NA	NA	✓	NA	NA	NA	NA	NA	NA	NA	NA	NA	x	NA	NA	x	x	NA	x	NA	1/7 (14.3)
	20b	NA	NA	NA	✓	✓	NA	NA	NA	✓	NA	NA	NA	NA	NA	NA	NA	NA	NA	✓	NA	NA	✓	✓	NA	✓	NA	7/7 (100.0)
	20c	NA	NA	NA	✓	✓	NA	NA	NA	✓	NA	NA	NA	NA	NA	NA	NA	NA	NA	✓	NA	NA	x	✓	NA	✓	NA	6/7 (85.7)
	20d	NA	NA	NA	✓	x	NA	NA	NA	✓	NA	NA	NA	NA	NA	NA	NA	NA	NA	x	NA	NA	x	x	NA	x	NA	2/7 (28.6)
Reporting biases	21	NA	NA	NA	✓	x	NA	NA	NA	✓	NA	NA	NA	NA	NA	NA	NA	NA	NA	x	NA	NA	x	x	NA	x	NA	2/7 (28.6)
Certainty of evidence	22	x	x	x	x	x	x	x	x	x	x	x	x	x	x	x	x	x	x	x	x	x	x	x	x	x	x	0/26 (0.0)
Discussion																												
Discussion	23a	✓	✓	✓	✓	✓	✓	✓	✓	✓	✓	✓	✓	✓	✓	✓	✓	✓	✓	✓	✓	✓	✓	✓	✓	✓	✓	26/26 (100.0)
	23b	✓	✓	x	✓	✓	✓	x	✓	✓	✓	✓	✓	✓	x	✓	✓	x	✓	x	x	✓	✓	x	✓	x	✓	18/26 (69.2)
	23c	x	x	x	✓	x	✓	x	✓	✓	x	x	x	x	x	✓	x	x	x	x	x	x	x	✓	✓	x	x	7/26 (26.9)
	23d	✓	✓	✓	✓	✓	✓	✓	✓	✓	✓	✓	✓	✓	✓	✓	✓	✓	✓	x	✓	✓	✓	✓	✓	x	✓	24/26 (92.3)
Other information																												
Registration and protocol	24a	x	x	x	x	x	x	x	x	x	x	x	x	x	x	x	x	x	x	x	x	x	x	x	x	x	x	0/26 (0.0)
	24b	x	x	x	x	x	x	x	x	x	x	x	x	x	x	x	x	x	x	x	x	x	x	x	x	x	x	0/26 (0.0)
	24c	x	x	x	x	x	x	x	x	x	x	x	x	x	x	x	x	x	x	x	x	x	x	x	x	x	x	0/26 (0.0)
Support	25	✓	✓	✓	✓	✓	✓	x	✓	✓	x	✓	✓	x	x	x	x	✓	✓	✓	✓	✓	✓	x	x	✓	✓	18/26 (69.2)
Competing interests	26	✓	x	✓	✓	✓	✓	✓	✓	✓	x	✓	✓	✓	x	✓	✓	✓	✓	✓	✓	✓	✓	x	x	✓	✓	21/26 (80.8)
Availability of data, code and other materials	27	x	x	x	x	x	✓	✓	✓	x	x	x	x	x	x	x	✓	x	✓	x	x	x	x	x	x	x	✓	6/26 (23.1)
Number of satisfied items (n/n, %)		18/28 (64.3)	14/28 (50.0)	15/28 (53.6)	30/42 (71.4)	24/42 (57.1)	13/28 (46.4)	15/28 (53.6)	19/28 (67.9)	33/42 (78.6)	13/28 (46.4)	15/28 (53.6)	14/28 (50.0)	15/28 (53.6)	8/28 (28.6)	13/28 (46.4)	16/28 (57.1)	14/28 (50.0)	20/28 (71.4)	20/42 (47.6)	12/28 (42.9)	15/28 (53.6)	21/42 (50.0)	21/42 (50.0)	15/28 (53.6)	18/42 (42.9)	18/28 (64.3)	54.0 ± 10.6

*Watanabe et al., 2021, Krawczyk et al., 2022, Das et al., 2023, Dubey et al., 2023, Hasim et al., 2023a, and Hasim et al., 2023b were the only ones that performed meta-analyses, therefore items #12, #13a–13f, #14, #19, #20–21 were only applied in these studies.

**TABLE 3:** Quality assessment of reviews that are included *in vivo* studies (animal and human) using an instrument adapted from Assessment of Multiple Systematic Reviews (AMSTAR) – 2 (Rocque et al., 2021).

		AMSTAR-2 Items																
Studies	Year	1	2	3	4	5	6	7	8	9	10	11	12	13	14	15	16	Overall rating
Popoola JO	2020	1	0	0.5	0.5	1	0	1	0.5	0.5	0	NM	NM	0.5	0	NM	1	6.5/16
Phimarn W	2021	1	0	1	0.5	1	0	1	1	0.5	0	0.5	1	1	0.5	1	1	11/16
Krawczyk M	2022	1	0	0.5	0.5	1	1	0.5	0.5	0	0	1	0.5	1	0.5	0	1	9/16
Louisa M	2022	1	0	0.5	1	0	1	1	1	0	0	NM	NM	0.5	0	NM	1	7/16
Watanabe S	2021	1	0	0.5	0.5	0	1	1	1	1	0	1	1	1	1	1	1	12/16
Karimi I	2022	1	0	0.5	0	0	0	1	0	0	0	NM	NM	0	0	NM	0	2.5/16
Redha AA	2021	1	0	0.5	0.5	0	0	1	1	0	0	NM	NM	0	0.5	NM	1	5.5/16
Nova E	2020	1	0	0.5	0	0	0	0.5	1	0	0	NM	NM	0	0.5	NM	1	4.5/16
Nurul M	2020	1	0	0	0.5	0	0	1	0	0	0	NM	NM	0	0	NM	0	2.5/16
Da Silva MG	2022	1	0	0	0.5	1	0	1	0	0	0	NM	NM	0	0	NM	1	4.5/16
Nurhayati T	2024	1	0	0.5	0.5	1	1	1	0.5	1	0	NM	NM	1	0	NM	1	8.5/16
Setyani W	2023	1	0	0.5	0	0	0	0.5	0.5	0	0	NM	NM	0	0	NM	1	3.5/16
Salhab H	2023	1	0	0.5	0.5	1	0	1	0.5	0	0	NM	NM	1	0	NM	1	6.5/16
Das M	2023	1	0	0.5	0.5	0	0	1	0.5	0	0	0	0	0	0	0	1	4.5/16
Sivanesan RK	2022	1	0	0.5	0.5	0	1	1	1	0	0	NM	NM	1	1	NM	0	7/16
Pasupuleti MK	2023	1	0	0	0.5	0	0	0.5	0.5	0	0	NM	NM	1	0	NM	1	4.5/16
TOTAL (%)	Yes	100.0	0	6.25	6.25	37.5	31.25	75.0	43.75	12.75	0	12.5	12.5	43.75	12.5	12.5	81.25	-
	Partial yes	0	0	75.0	75.0	0	0	25.0	37.5	12.75	0	6.25	6.25	12.5	25	0	0	-
	No	0	100.0	18.75	18.75	62.5	68.75	0	18.75	75	100	6.25	6.25	43.75	62.5	12.5	18.75	-
	NM	0	0	0	0	0	0	0	0	0	0	75	75	0	0	75	0	-

The AMSTAR-2 Items scored as 0, 0.5 or 1. High methodological quality: ≥12/16 and Low methodological quality: ≤7/16. NM: No meta-analyses.

Each item in the PRISMA 2020 checklist was evaluated for the included reviews (Table 2). Of the 42 items included in the PRISMA 2020 Statement, only 15 were satisfied by greater than 80% of the studies: Title (Yatoo et al., 2018), Introduction (Khumalo et al., 2022; Pahwa et al., 2023), Methods (7, 12, 13a, 13d), Results (16a, 17, 19, 20b, and 20c), Discussion (23a and 23d) and Other information (Aumeeruddy and Mahomoodally, 2021). Items 2, 15, 22 and 24a-24c were not reported in any included SR. Among the Meta-analyses exclusive items, numbers 12, 13a, 13d (Methods) and 19, 20b, 20c (Results) were the most reported. The completion percent mean for all included SRs (n = 26) within the PRISMA checklist was 54.0% [standard deviation (SD) = 10.6%], with the article by Watanabe et al. (2021), Hasim et al. (2023a) being the highest (78.6%) and the by Nurul and Harun (2020), (Hasim et al., 2023b) the lowest (28.6%) adherence to Statement.

**FIGURE 7:**
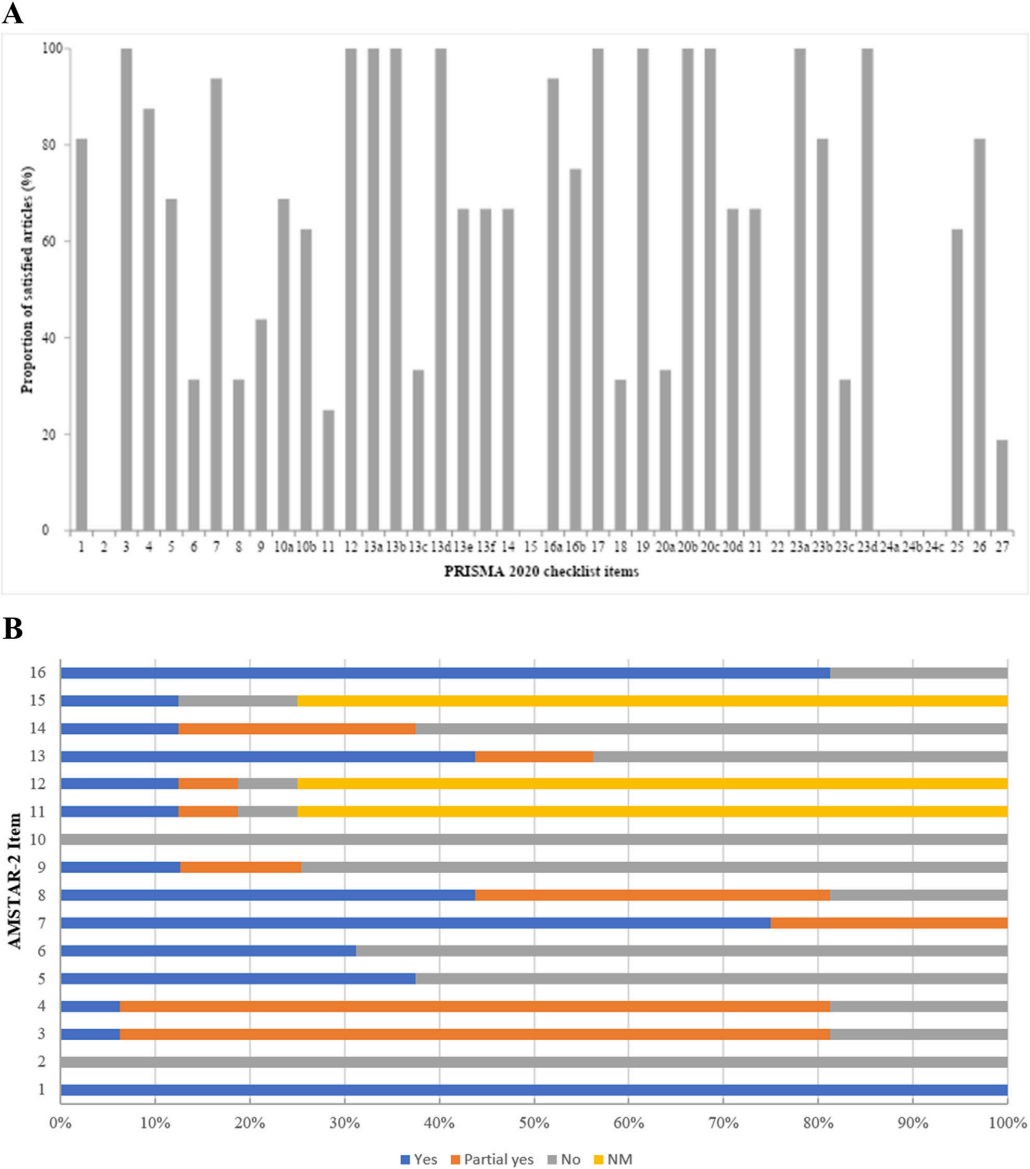
Bar chart of the proportion of articles that satisfied each item of the PRISMA 2020 checklist **(A)** and AMSTAR 2 **(B)**.

For AMSTAR-2 analysis, we excluded ten SRs from our original sample size, since our instrument was adapted to analyze *in vivo* interventions (animals and humans), and thus, this assessment included 16 SRs (Table 3). The most commonly fully satisfied criteria (≈80%) were #1 (Population, Intervention, Comparator, Outcome (PICO) components) with 16/16 (100%) of included systematic reviews fully satisfying this criterion, #7 (inclusion and exclusion criteria explanation) (12/16 = 75.0% fully and 4/16 = 25.0% partially) and #16 (potential sources of conflict of interest reported) (13/16 = 81.25% fully and 2/16 = 18.75% not) (Figure 7). None of the selected studies reported the pre-existence of a protocol (#2) and sources of funding for individual studies (#10). For criteria #11, #12, and #15, which only applied to reviews including meta-analyses, 2/4 (50.0%) fully satisfied criterion #11 (use of an appropriate methods for statistical combination of results) (1/4 = 25.0% partially), 2/4 (50.0%) fully satisfied criterion #12 (assessment of the potential impact of Risk of Bias (RoB) or limitations in individual studies) (1/4 = 25.0% partially), and 2/4 (50.0%) fully satisfied criterion #15 (an adequate investigation of publication bias, small study bias) (2/4 = 50.0% not). The quality scores ranged from 2.5/16 to 12/16 with one higher quality review (score ≥12/16) (Watanabe et al., 2021) and twelve lower quality reviews (scoring 7/16 or less) (Nova et al., 2020; Ali Redha et al., 2021; Popoola et al., 2020; Louisa et al., 2022; Pasupuleti et al., 2023; Da Silva et al., 2022; Nurul and Harun, 2020; Salhab et al., 2023; Das et al., 2023; Sivanesan et al., 2022; Setyani et al., 2023; Karimi, 2022).

**FIGURE 8:**
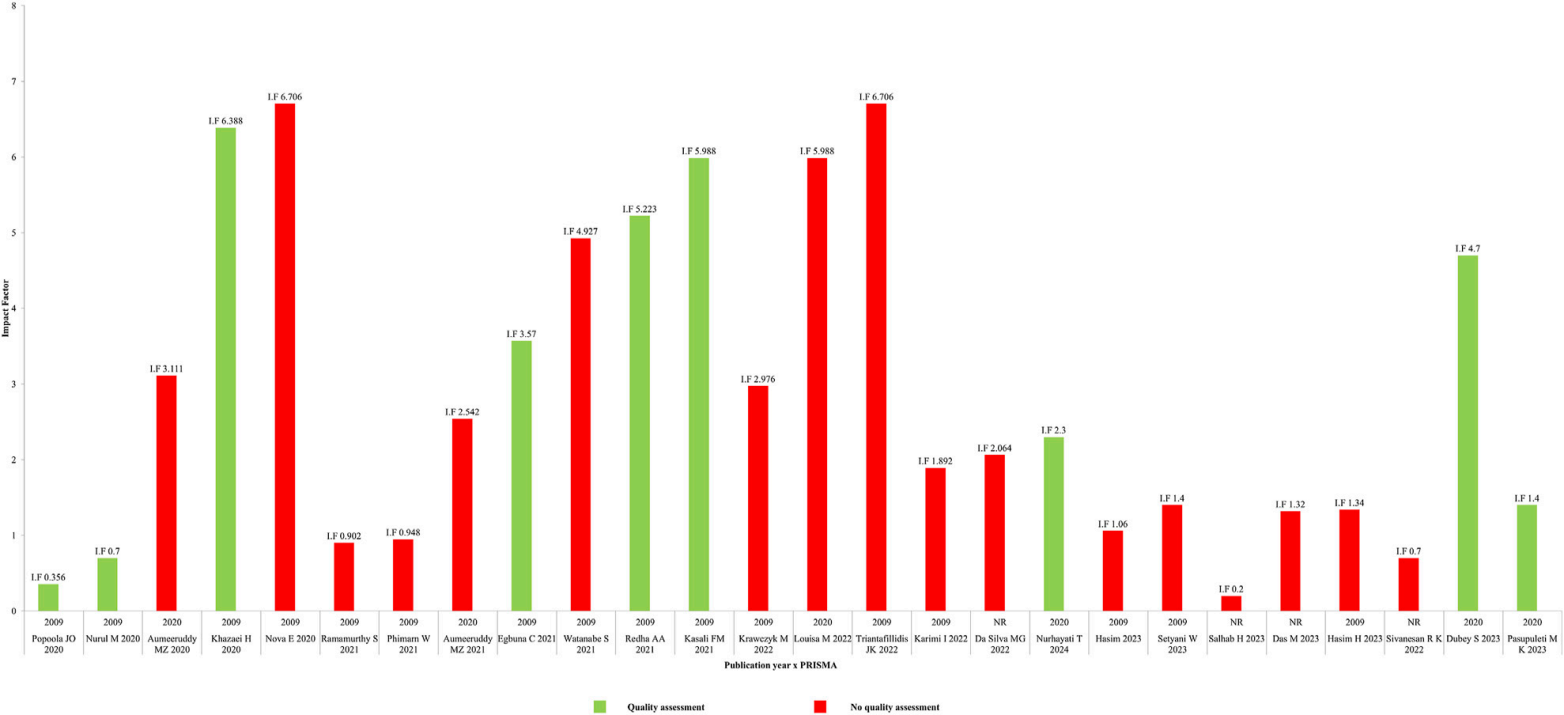
Quality assessment evaluation. Relationship between the impact factor of the journals where the SRs were published, the year of publication, the PRISMA checklist applied (2009 or 2020) and the tools used to assess the methodological quality of individual studies included in each SR.

An additional evaluation was carried out to assess the relationship between the impact factor of the journals where the SRs were published, the year of publication, the PRISMA checklist applied (2009 or 2020) and the tools used to assess the methodological quality of individual studies included in each SR (Figure 8). The scientific quality was assessed in eight SR: three studies used the Cochrane Risk of Bias Assessment Tool (Watanabe et al., 2021; Pasupuleti et al., 2023; Ramamurthy et al., 2021), the fourth used Camarades and Jadad scale (Kasali et al., 2021), the fifth used Cochrane Risk of Bias Assessment Tool and Jadad scale (Phimarn et al., 2021), the sixth (Popoola et al., 2020) used a procedure by Siqueira-Lima et al. (2017), the seventh used Joanna Briggs Institute (JBI) Critical Appraisal Checklist (Nurhayati et al., 2024), and the last one used a three-item five-point quality scale (Dubey et al., 2023). Through of Figure 8, it is possible to observe the use of quality tools is not related to the impact factor of the journals, since the article from the journal with the second lowest impact factor [(Popoola et al., 2020); I.F 0.356] performed this assessment, while the opposite did not [(Nova et al., 2020; Triantafillidis et al., 2022); I.F 6.706]. Also, through Figure 8, it is observed that even though the SRs were published between 2020 and 2024, the majority (n = 16; 61.53%) still use the oldest version of PRISMA (2009). Moreover, the SRs that were based on the new version of PRISMA (2020) did not perform quality assessment (Aumeeruddy and Mahomoodally, 2020; Aumeeruddy and Mahomoodally, 2021; Hasim et al., 2023a; Hasim et al., 2023b; Da Silva et al., 2022; Salhab et al., 2023; Setyani et al., 2023; Das et al., 2023; Sivanesan et al., 2022; Dubey et al., 2023).

## 4 Discussion

### 4.1 Characteristics of included studies

Globally, there is increased scientific research, commercialization, utilization and demand for MO based pharmaceutics and supplements to challenge various illnesses and support healthier living. MO-based products and supplements are becoming increasingly available in the open market, as well as online portals, in various forms of presentation (vegetable drug, flours, oils, supplements) and disclosed with different therapeutic indications, not allowed for food, which would characterize them as phytotherapics, and without commensurate toxicity assessment tests to establish their safe consumption (Popoola et al., 2020).

From the year 2000 up until 6 August 2020, there were 2,345 published research papers on the MO as indexed on the Scopus database. In the last 5 years (2016–2020) research outputs grew by 50%. The number of research outputs during this period indicates an elevated interest in the plant (George et al., 2021). Among these publications, several SRs or MAs have investigated the efficacy and safety of MO for treating certain conditions, however there is no umbrella review in the literature, which simultaneously covers all these conditions. To our knowledge, this is the first umbrella review of SRs and MAs of clinical trials, and experimental *in vitro* or *in vivo* studies that evaluated the effect of MO on inflammatory diseases.

Our findings indicated that the effects of MO on inflammatory diseases have been a great research of interest especially among Asian, African, and European researchers. Besides, the fact that the primary articles originate mainly from Africa, Asia, Europe, and America is an indication of global research interest on MO. Moreover, Indian researchers were the greatest investigators into the use and exploitation of MO probably because India is a region native to this plant and because it is known for practicing traditional herbal medicine for the health needs of the primary population.

Different parts of the plant have varying bioactive phytochemicals and biological potency. As shown in Figure 4A, the MO leaves were the most used parts of the plant. The high use of leaves may be due to their richness in active constituents, their high abundance and availability, in addition to the ease of collection. In terms of conservation, the high use of leaves can also be explained by indicating a more sustainable practice when compared to other parts of the plant, for example, the root harvesting process can be a threat to the survival of some species and excessive use of fruits and seeds can cause adverse effects on the genetic diversity and distribution of plants due to their role in sexual reproduction and dispersal (Karimi, 2022).

Plants require different extraction conditions to achieve maximum recovery of bioactive phytochemicals, which makes the extractive process a key process in herbal medicines (Aumeeruddy and Mahomoodally, 2021). In this overview, it was observed that decoction was the main extraction method used (Figure 4B). The high use of the decoction is probably because of heat that accelerates the extraction process, as it accelerates biological reactions and increases the availability of bioactive compounds. However, decoction is only efficient for the extraction of thermostable constituents and for MO, although decoction was the most used extractive process among the SRs included, heat treatment seemed to reduce the pharmacological effect associated with glucosinolate metabolites, which need to be hydrolyzed into isothiocyanates to become bioactive. Hot teas prevent this hydrolysis due to inactivation of the heat-sensitive enzyme. So, cold water tea preparations seemed to be preferable, since they can provide enough glucosinolates and their isothiocyanate metabolites for potential biological activity, as they contain myrosinase, the enzyme responsible for the conversion of glucomoringin to isothiocyanate moringin (Nova et al., 2020; Ramamurthy et al., 2021).

A few SRs included the types of MO formulation used in the clinical trials (Supplementary Data S3B). Ingestion of encapsulated powder, in tablets or added with a meal were most often used as a method of preparation. It is known that the content of bioactive compounds can vary depending on the cultivation conditions, geographic origin, harvest time, soil and climate factors, process of extraction and storage. These variables make it difficult to have standardized and reliable raw materials (Silva et al., 2022; Watanabe et al., 2021). It is through the extractive process that it is possible to isolate or concentrate and standardize the content of active ingredients. Studies rarely reported the yield and contents of their extracts, among the clinical trials, only one primary study (Nambiar et al., 2010) included in the SR of Popoola et al. (2020) reported the content of active constituent in MO extract used, 3,700 µg of beta carotene and 1775 mg of total phenols in the SR. Therefore, despite the extensive evidence of the beneficial properties of MO, standardization regarding the content of bioactive compounds and quality control is still needed in the manufacture of these preparations.

The different physicochemical attributes of the extracts and phytocompounds of interest dictate us to select the most suitable solvents for the extraction or fractionation of part of the MO to obtain more potent formulations or even the isolated phytocompound itself (Karimi, 2022). Moreover, the type of solvent used for extraction can significantly affect the extraction of bioactive compounds and thus bioactivity. An example of this is shown in the studies by Swamy and Meriga (2020) and Adisakwattana and Chanathong (2011), which analyzed the inhibition of pancreatic lipase by hydroalcoholic and aqueous extracts of MO leaves, respectively (Ali Redha et al., 2021). It was observed that the hydroalcoholic extract inhibited the enzyme, while the aqueous extract did not.

In this overview, water and ethanol were the most used solvents in the SRs included (Supplementary Data S3B). Ethanolic extracts were more potent than aqueous extracts in *in vitro* studies, possibly due to the preferential concentration or isolation of active components during the extraction process (Ramamurthy et al., 2021), and due to ethanol being a biocompatible solvent and extract. Polyphenols power due to the different polarities of bioactive phytochemical constituents (Silva et al., 2022). It was observed that typical organic solvents were also used to prepare MO extracts. However, herein, we are focused on future pharmacological applications of MO extracts, so the solvents methanol, n-hexane, ethyl acetate, chloroform, petroleum ether should be avoided, as they are toxic and are not interesting for the formulation of phytotherapics.

In view of the analysis carried out in this overview, it was observed that the phytochemicals mentioned in the SRs included were rarely related to the type of solvent used in the extract, therefore, the main bioactives present in the MO were analyzed through the production of word clouds. Word clouds are images composed of main terms related to a particular subject, in which the size of each word indicates its frequency or relevance. It was observed that the most relevant bioactive compounds involved in the anti-inflammatory, antioxidant, anti-obesity, hypoglycemic and antitumor activities of MO were flavonoids, phenolic acids and isothiocyanates.

Based on the available evidence, MO presents a relatively favorable safety profile when consumed in moderate doses, with clinical studies showing no significant increase in adverse events at doses of up to 4 g Nova et al., 2020. Histopathological tests, relative organ weights, and toxicity biomarkers did not indicate any relevant adverse effects, and both aqueous and methanolic extracts were shown to be safe in rodent studies, even at high doses (up to 6,400 mg/kg) (Popoola et al., 2020). However, some studies reported mild side effects such as frequent urination, headache, cough, and changes in urine color, although these effects were observed in only two studies (Phimarn et al., 2021). There is also evidence that high consumption levels (40–60 g) may lead to changes in hematological parameters and increased cholesterol levels (Louisa et al., 2022). Furthermore, Moringa may interfere with the action of drugs metabolized by the cytochrome P450 enzyme system, and caution is advised when used concomitantly with prescription medications (Triantafillidis et al., 2022).

### 4.2 Methodological concerns

The methodological quality of an SR represents how well it was conducted. It is widely recognized that high-quality SRs play a crucial role in consolidating the evidence base. Well-conducted reviews increase the likelihood of presenting unbiased results and are a prerequisite for valid interpretations and applications. It is important to recognize that methodological flaws can have a significant impact on the results and conclusions of SRs, which can lead to reliability and reproducibility issues (Zoltowski et al., 2014; Bodnaruc et al., 2024).

The SRs show promising effectiveness of MO for diabetes mellitus, obesity, cancer, hypertension, dyslipidemia, among other inflammatory diseases, but the quality of these SRs is questionable. It is known that in 2009 the first Preferred Reporting Items for Systematic reviews and Meta-Analyses (PRISMA) statement was published with the aim of improving the quality of reports. Since then, methodological approaches, such as result synthesis and risk of bias assessment, have advanced, requiring updated guidelines; thus, an updated version of the PRISMA statement was published in 2020 (Park et al., 2022). Although all 26 SRs included in this review were published after 2020, only six of them (Aumeeruddy and Mahomoodally, 2020; Aumeeruddy and Mahomoodally, 2021; Louisa et al., 2022; Pasupuleti et al., 2023; Dubey et al., 2023; Nurhayati et al., 2024) indicated that they followed this latest version of PRISMA (2020), by citing the 2020 reference and/or using the flow diagram adopted in the 2020 version, and, nevertheless, they did not reach even 80% of compliance with the checklist in our evaluation. It is worth highlighting that the instrument of choice for evaluating the methodological quality of SRs is the AMSTAR-2. This is a 16-domain tool, applicable to SRs that include randomized and/or non-randomized controlled trials. We adapted it, from Rocque et al. (2021), to suit a research context that is not amenable to controlled trials, and thus it was possible to evaluate and classify the SRs, predominantly, as of low methodological quality (≤7/16), there may be a greater likelihood of ambiguous and inconsistent results regarding the efficacy and safety of *M. oleifera*. Therefore, it is suggested that future studies should comply with the PRISMA guideline and AMSTAR 2 to improve the methodology quality of SRs about MO.

### 4.3 Inconsistency in outcomes

A lack of consistency was observed between the results of some outcomes, which reduces the scientific evidence for these findings. This inconsistency can be explained by numerous factors related to the experimental designs used. Among studies, different types of MO were used, such as MO extract, MO powder, or MO capsule. The treatment dose and duration of MO among studies were also various. Among animal studies, different chemicals were used to induce diabetes, at different rodent’s age and periods of treatment. In human studies, a limited number of patients were used, lack of standardized preparations and doses of MO extract. Furthermore, the different origin of the MO plants, genetic background, soil, climate, season, and the use of different procedures of processing and storage and the extraction methods employed can also influence the underlying mechanisms due to the different composition of the plant and extracts (Nova et al., 2020; Louisa et al., 2022).

### 4.4 Mechanisms of action

Regarding the mechanism of action, some studies have been reported, mainly those related to antidiabetic activity. The antidiabetic activity of this plant may be the result of alleviating insulin resistance, either by neutralizing oxidative stress or by attenuating inflammation. Several anti-diabetic pharmacological mechanisms have been suggested in MO extract, including the stimulation of insulin secretion, inhibition of α-amylase and α-glucosidase activities, decrease of gluconeogenesis in the liver, improve glycogen synthase activities, increase of glucose uptake in the muscles and liver, inhibition of glucose uptake from the intestine. MO restores the activities of hexokinase (HK) and glucose 6-phosphate dehydrogenase (G6PD), facilitating glycolysis and glucose utilization by the pentose phosphate pathway. When it comes to insulin sensitivity, MO acts by stimulating the insulin-dependent Akt pathway, upregulating glucose transporter GLUT4 expression in the muscles, and by increasing the expression of insulin receptor and insulin receptor substrate 1 in the liver. Through its antioxidant activity, MO decreases expression of pyruvate carboxylase enzymes in the liver and regenerates damaged pancreatic β-cells and hepatocytes. Furthermore, the high fiber content in MO leaves can improve glycemic control in the postprandial state due to delayed gastric emptying (Nova et al., 2020; Krawczyk et al., 2022; Phimarn et al., 2021; Watanabe et al., 2021; Setyani et al., 2023).

The multitude of mechanisms that underlie the dyslipidemia effects of MO mostly include inhibition of β-hydroxy β-methylglutaryl-CoA (HMG-CoA) reductase, and enhanced endocytosis of low-density lipoprotein cholesterol (LDL) by activation of LDL receptor. In addition, other mechanisms are associated with anti-obesity activity: inhibition of pancreatic lipase, inhibition of expression of adipogenesis associated proteins (PPARy and FAS), increase of expression of Lipolysis-associated protein (ATGL), increase the levels of ghrelin, and decrease the secretion of leptin. Furthermore, in adipose tissues, MO was shown to normalize increased mRNA levels of leptin and resistin, and decreased those of adiponectin, melanocortin receptor-4, and peroxisome proliferator-activated receptors (Krawczyk et al., 2022; Ali Redha et al., 2021; Phimarn et al., 2021; Sivanesan et al., 2022).

The pharmacodynamics associated with the anticancer activity of MO include the induction of apoptosis through of the activation of the tumor suppressor p53, targeting extrinsic and intrinsic pathways, and of ROS-mediated signaling pathway, the anti-angiogenesis action through the inhibition of NF-kB signaling, the anti-proliferation action, through inhibition of microtubule assembly and of activation of cell cycle arrest at G2/M phase, the anti-inflammation action through inhibition of COX and NO, of MAP-kinase family and of NF-kB signaling, and the transformation of carcinogen through of inhibition of CYPs (inactivate carcinogenesis) and of carcinogen detoxification (activation of GST and NQO1, of NFR2-ARE pathway, and of NRF2 pathway) (Salhab et al., 2023; Karimi, 2022; Soto et al., 2025).

**FIGURE 9:**
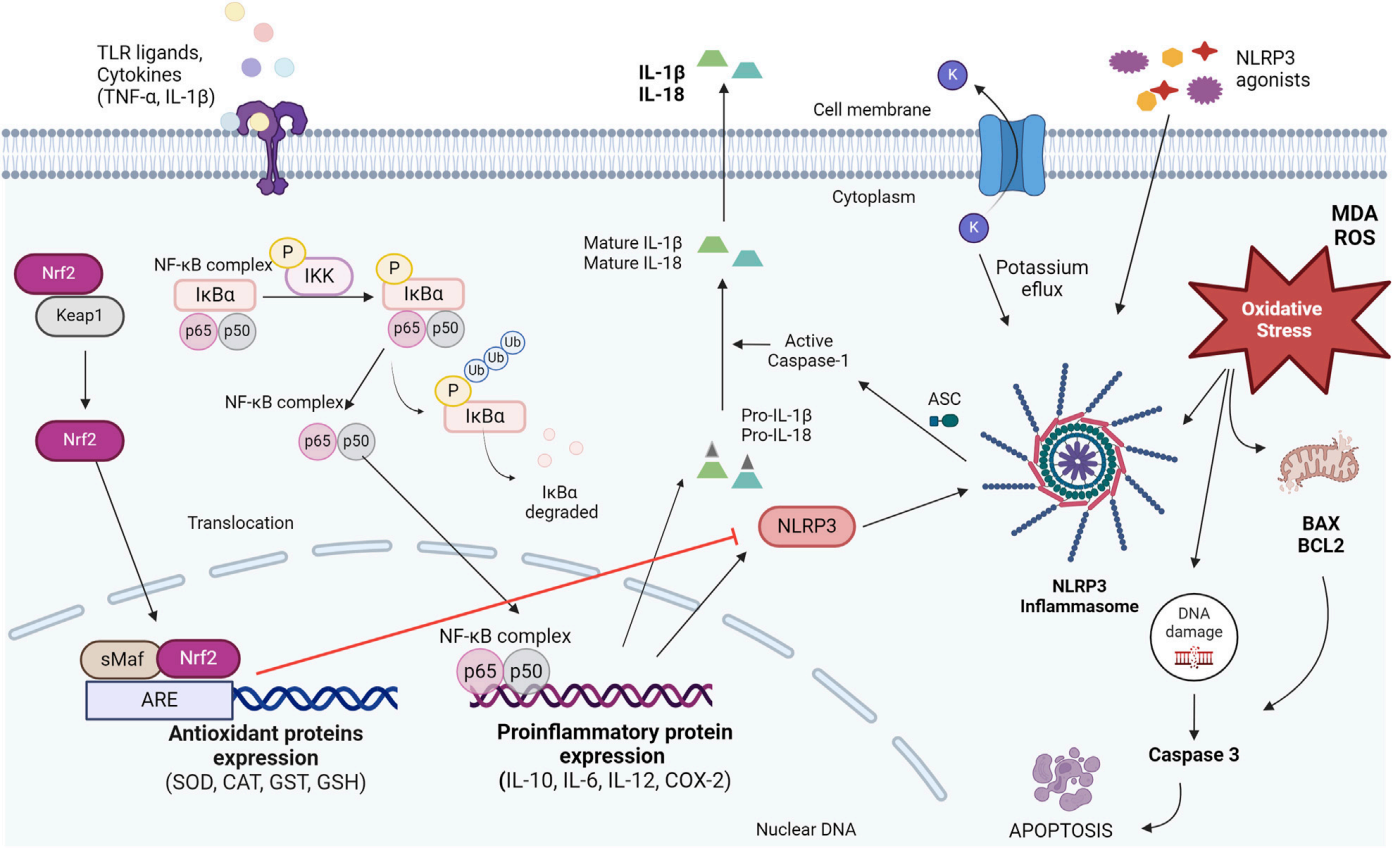
Oxy-inflammation mechanism, Nrf2 and NF-κB signaling pathway and regulation of the NLRP3 inflammasome. *Moringa oleifera* is an inhibitor of IL-1β production mediated by the NLRP3 inflammasome, acting on more than one element of the pathways involved (inhibition of IκBα phosphorylation and degradation; inhibition of translocation of the NF-κB complex to the nucleus, inhibition of NF-κB expression) and an upregulator of Nrf2 signaling. Nrf2 negatively regulates the NLRP3 inflammasome. ASC, apoptosis-associated Speck-like protein; ARE, antioxidant response elements; CAT, catalase; GSH, glutathione; GST, glutathione transferase; IKK, IκB kinase complex; IκBα, NF-κB inhibitor; IL-10, interleukin 10; IL-6, interleukin 6; IL-12, interleukin 12; COX-2, cyclooxygenase 2; IL-1β, interleukin 1β; IL-18, interleukin 18; Keap1, Kelch-like ECH-associated protein; MDA, malondialdehyde; NF-κB, nuclear factor- κB; NLRP3, NLR (nucleotide-binding leucine-rich repeat receptor) family pyrin domain containing 3; Nrf2, NF-E2 p45-related factor 2; ROS, reactive oxygen species; sMaf, small Maf proteins; SOD, superoxide dismutase; TNF-α, tumor necrosis factor alpha; TLRs, toll-like receptors. Elaborated using an online platform for creating scientific illustrations (BioRinder).

The possible pathways of action of MO include the modulation of anti-inflammatory and antioxidant signaling. Many studies imply that one of the anti-inflammatory mechanisms of MO is routed *via* the vital NF-κB pathway. MO suppressing NF-κB protein and its translocation to the nucleus, which resulted in downregulation of pro-inflammatory genes. In addition, MO upregulates Nrf2, which resulted in the increased transcription of anti-oxidative and cytoprotective genes, and anti-inflammatory cytokines (Louisa et al., 2022; Watanabe et al., 2021). Therefore, MO may possibly act on its oxy-inflammation mechanism through modulation of inflammasomes, as suggested in Figure 9.

The main phytochemical compounds extracted from the leaves of MO include glucosinolates, flavonoids and phenolic acids that have a protective effect against chronic diseases through different mechanisms. Polyphenols may inhibit protein oxidation, formation of AGEs, and protein cross-linking in glycation reactions. Regarding flavonoids, it has been reported that they can inhibit glucose transporter proteins in cell membranes inhibiting the intestinal glucose uptake, inhibit arachidonic acid and lysosomal enzyme secretion from the endothelial thereby inhibiting the inflammatory process, and activate rat paraoxonase 1 (rPON1) and catalase (rCAT) activities. Several mono-glucosides of quercetin and kaempferol have a strong binding ability to bind to the enzymes α-amylase and α-glucosidase, inhibiting them. The phenolic acids gallic, chlorogenic, and caffeic acids also have an inhibitory effect on these enzymes. Quercetin, kaempferol and chlorogenic acid act as competitive inhibitors of the SGLT1 in the small intestine, reducing the absorption of glucose. Quercetin can act as an inhibitor of GLUT2, may activate adenosine monophosphate-activated protein kinase (AMPK) to increase glucose uptake through stimulation of GLUT4 in skeletal muscle and to decrease the production of glucose through downregulation of phosphoenolpyruvate carboxykinase (PEPCK) and glucose-6 -phosphatase (G6Pase) in the liver, and can inhibit the Na+ -dependent glucose uptake *via* the SGLT-1 transporter. Finally, isothiocyanates, active molecules derived from the hydrolysis of glucosinolates, appear to be involved in glycemic control due to their ability to reduce resistance to the action of insulin and hepatic gluconeogenesis (Nova et al., 2020; Krawczyk et al., 2022; Watanabe et al., 2021; Nurul and Harun, 2020; Setyani et al., 2023).

An overview of systematic reviews has the main advantage of providing a wide range of perspectives on the intervention and their relative efficacy (Yuan et al., 2017). However, the present overview may have some limitations, including a possible missed relevant review, although our search strategy appeared complete. Moreover, many of the primary studies were included in more than one review among the selected articles and no assessment of overlap was performed, which could influence the results of this umbrella review. It is also possible that the evaluation of the SRs, rather than the original RCTs, did not capture the relevant details from the primary studies. Furthermore, we did not assess the quality of primary studies as this was not within the scope of this review.

## 5 Conclusion

It was possible to observe that *M. oleifera* is a promising plant with great potential for the treatment of inflammatory diseases and that it possibly acts on the oxy-inflammation mechanism through the NF-κB and Nrf2 pathways with modulation of the inflammasome. However, despite the evidence reported in this review, the translation of the biological potential of moringa into clinical practice has not yet been achieved, a fact that is evidenced by the scarcity of approved clinical trials in studies related to this plant.

Based on the results obtained in this review, it was possible to find several limitations that should be considered before applying the plant as a medicine. The source of *M. oleifera* used in the experiments was not homogenized; both the raw plant (leaf, flower, seed, fruit, *etc.*) and plant derivatives were used as interventions. Some articles included in the review reported the use of raw leaf powder, without outlining any extraction protocol for the study. Furthermore, when the object of study was the extract, it was observed that the solvent used for plant extraction was also not standardized, varying between methanol, ethanol and water. These different sources of origin, as well as the lack of standardization of extracts, can produce discrepancies in phytochemical content and, therefore, different effects, which was evidenced in this review, since a wide variation was observed in the outcomes analyzed within the same experimental model and also when comparing clinical trials with preclinical trials.

Therefore, in order to ensure consistent therapeutic results, the need for standardized cultivation and extraction protocols is highlighted. However, it is not possible to suggest a standardization method for *M. oleifera* in our study, since the quality control of a herbal medicine requires an official pharmacopoeial monograph for the plant, which does not exist for *M. oleifera*. This means that there is no specific marker or quality standard to be followed for the plant raw material or its derivatives. Accordingly, to better standardize and understand the results generated by future studies, it would be essential to develop a pharmacopoeial monograph for the species.

Although several *in vitro* and *in vivo* studies analyzed have demonstrated safety and efficacy, it is not possible to extrapolate these findings to the human population, and more robust and well-controlled research is necessary. In addition, this review found enormous heterogeneity in the design of the experiments performed, where it was observed that the vast majority presented a lack of standardization in their protocol (non-standardization of the dose, pharmaceutical form used and type of extraction, use of plant powder instead of extract, lack of identification and quantification of phytochemical markers, among others).

In the face of all this, to ensure consistent therapeutic results, future studies should evaluate the safety and efficacy of standardized extracts in a sufficient number of patients and in the long term, adequately specifying information on (i) detailed method of preparation, (ii) dose, frequency and duration of treatment and (iii) positive results or adverse effects. Additionally, in order to improve quality, future SRs should comply as much as possible with the PRISMA and AMSTAR 2 tools.

## Data Availability

The original contributions presented in the study are included in the article/Supplementary Material, further inquiries can be directed to the corresponding author.
