# Supplementary material for: Effect of Moringa oleifera on inflammatory diseases: an umbrella review of 26 systematic reviews
Source: Front Pharmacol. 2025 May 19;16:1572337. doi: 10.3389/fphar.2025.1572337 (PMC12127422; doi:10.3389/fphar.2025.1572337)
Supplement: Supplementary file 1 [file Table1.docx]

**Supplementary data 1:** Full search strategy in Embase, Scopus, Web of Science, PubMed and Cochrane library including search terms and filters.

| **Data base** | **Descriptors** | **Items Found** | **Time** | **Date** |
| --- | --- | --- | --- | --- |
| **E**  **M**  **B**  **A**  **S**  **E** | #Filter 1: Moringa oleifera  “moringa oleifera” OR “drumstick tree” OR horseradish | 36,271 | 8:33 | 04/11/2024 |
|  | # Filter 2: Systematic Review and Meta-analysis  “systematic review” OR “meta-analysis” | 794,706 | 8:36 | 04/11/2024 |
|  | **Total: #1 and #2** | 142 | 8:37 | 04/11/2024 |
| **Data base** | **Descriptors** | **Items Found** | **Time** | **Date** |
| **S**  **c**  **o**  **p**  **u**  **S** | #Filter 1: Moringa oleifera  (TITLE-ABS-KEY(“moringa oleifera”) OR TITLE-ABS-KEY(“drumstick tree”) OR TITLE-ABS-KEY(“horseradish”)) | 47,859 | 8:42 | 04/11/2024 |
|  | #Filter 2: Systematic Review and Meta-analysis  (TITLE-ABS-KEY(“Systematic Review”) OR TITLE-ABS-KEY(“Meta-analysis”)) | 777,239 | 8:44 | 04/11/2024 |
|  | **Total: #1 and #2** | 148 | 8:45 | 04/11/2024 |
| **Data base** | **Descriptors** | **Items Found** | **Time** | **Date** |
| **W**  **E**  **B**  **O**  **F**  **S**  **C**  **I**  **E**  **N**  **C**  **E** | #Filter 1: Moringa oleifera  TS=Moringa oleifera OR TS= drumstick tree OR TS= horseradish | 43,880 | 8:51 | 04/11/2024 |
|  | #Filter 2: Systematic Review and Meta-analysis: #2  TS= Systematic Review OR TS= Meta-analysis | 669,168 | 8:53 | 04/11/2024 |
|  | **Total: #1 and #2** | 53 | 8:54 | 04/11/2024 |
| **Data base** | **Descriptors** | **Items Found** | **Time** | **Date** |
| P  U  B  M  E  D | #Filter 1: Moringa oleifera  ("moringa oleifera"[MeSH Terms] OR moringa oleifera[TIAB] OR moringa oleifera[TIAB] OR drumstick tree[TIAB] OR horseradish[TIAB]) | 25,974 | 8:59 | 04/11/2024 |
|  | #Filter 2: Systematic Review and Meta-analysis  ("systematic review"[Publication Type] OR "systematic reviews as topic"[MeSH Terms] OR "meta-analysis"[Publication Type] OR "meta-analysis as topic"[MeSH Terms] OR "meta-analysis"[TIAB]) | 464,867 | 9:00 | 04/11/2024 |
|  | **Total: #1 and #2** | 19 | 9:01 | 04/11/2024 |
| **Data base** | **Descriptors** | **Items Found** | **Time** | **Date** |
| C  O  C  H  R  A  N  E  L  I  B  R  A  R  Y | #Filter 1: Moringa oleifera  (moringa oleifera) OR (drumstick tree) OR (horseradish) | 231 | 9:46 | 04/11/2024 |
|  | #Filter 2: Systematic Review and Meta-analysis  (systematic review) OR (meta-analysis) | 50,675 | 9:47 | 04/11/2024 |
|  | **Total: #1 and #2** | 8 | 9:47 | 04/11/2024 |
