# Supplementary material for: Effect of Moringa oleifera on inflammatory diseases: an umbrella review of 26 systematic reviews
Source: Front Pharmacol. 2025 May 19;16:1572337. doi: 10.3389/fphar.2025.1572337 (PMC12127422; doi:10.3389/fphar.2025.1572337)
Supplement: Supplementary file 2 [file Table2.docx]

**Supplementary data 2**

**AMSTAR-2 (adapted)**

**1. Did the research questions and inclusion criteria for the review include the components of PICO?**

For Yes:

( ) Population

( ) Intervention

( ) Comparator group (if applicable)

( ) Outcome (if applicable)

Optional:

( ) Yes

( ) No

( ) Timeframe for follow-up

**2. Did the report of the review contain an explicit statement that the review methods were established prior to conduct of the review?**

For Yes:

( ) A protocol must have been established before the review.

( ) Yes

( ) No

**3. Did the review authors explain their selection of the study designs for inclusion in the review?**

For Partial Yes:

( ) The study designs are specified

For Yes:

( ) The study designs are explained.

( ) Yes

( ) Partial Yes

( ) No

**4. Did the review authors use a comprehensive literature search strategy?**

( ) Yes

( ) Partial Yes

( ) No

For Partial Yes (all the following)

( ) searched at least 2 databases (relevant to research question)

( ) provided key word and/or search strategy

For Yes (all the following):

( ) justified publication restrictions (eg, language)

( ) searched the reference lists/bibliographies of included studies

( ) included/consulted content experts in the field

( ) where relevant, searched for grey literature

( ) conducted search within 24 months of completion of the review

**5. Did the review authors perform study selection in duplicate?**

For Yes, either ONE of the following:

( ) at least two reviewers independently agreed on selection of eligible studies and achieved consensus on which studies to include.

( ) Yes

( ) No

( ) OR two reviewers selected a sample of eligible studies and achieved good agreement (at least 80 per cent), with the remainder selected by one reviewer.

**6. Did the review authors perform data extraction in duplicate?**

For Yes, either ONE of the following:

( ) at least two reviewers achieved consensus on which data to extract from included studies.

( ) Yes

( ) No

( ) OR two reviewers extracted data from a sample of eligible studies and achieved good agreement (at least 80 per cent), with the remainder extracted by one reviewer.

**7. Did the review authors provide an explanation of the inclusion and exclusion criteria.**

( ) Yes

( ) Partial Yes

( ) No

For Partial Yes:

( ) There is only one out, explanation of the inclusion OR exclusion criteria

For Yes:

( ) The two, inclusion and exclusion criteria, must be explained.

**8. Did the review authors describe the included studies in adequate detail?**

For Partial Yes (all the following)

( ) described populations

( ) described interventions

( ) described comparators (if applicable)

( ) described outcomes (if applicable)

( ) described research designs

For Yes (all the following):

( ) described populations in detail

( ) Yes

( ) Partial Yes

( ) No

( ) described interventions in detail (including doses where relevant)

( ) described outcomes (if applicable) in details

( ) described study’s setting

( ) timeframe for follow-up

**9. Did the review authors use a satisfactory technique for assessing the risk of bias (RoB) in individual studies that were included in the review?**

**9.1 If the paper contains ONLY clinical studies, consider original evaluation:**

**RCTs**

For Partial Yes, must have assessed RoB from:

( ) unconcealed allocation, AND

( ) lack of blinding of patients and assessors when assessing outcomes (unnecessary for objective outcomes such as all cause mortality).

For Yes, must also have assessed RoB from:

( ) Yes

( ) Partial Yes

( ) No

( ) Includes only NRSI

( ) allocation sequence that was not truly random, AND

( ) selection of the reported result from among multiple measurements or analyses of a specified outcome.

**NRSI**

For Partial Yes, must have assessed RoB:

( ) from confounding, AND

( ) from selection bias.

For Yes, must also have assessed RoB:

( ) Yes

( ) Partial Yes

( ) No

( ) Includes only RCTs

( ) methods used to ascertain exposures and outcomes, AND

( ) selection of the reported result from among multiple measurements or analyses of a specified outcome.

**9.2. If the paper contains clinical and NON-clinical trials (*in vivo*), consider:**

**For clinical trials:**

**RCT:**

( ) Yes

( ) No

( ) Includes only NRSIs

For Yes, must have assessed RoB from

( ) unconcealed allocation, AND

( ) lack of blinding of patients and assessors when assessing outcomes (unnecessary for objective outcomes such as all cause mortality).

( ) Yes

( ) No

( ) Includes only RCTs

**NRSI**

For Yes, must have assessed RoB:

( ) from confounding, AND

( ) from selection bias.

**For Non-clinical trials:**

( ) Yes

( ) No

( ) Instead of assessing the RoB, the review authors must have used a satisfactory technique for assessing the limitations in individual studies that were included in the review.

**Total Result:**

( ) Yes

( ) Partial Yes

( ) No

For Partial Yes:

( ) If there is at least one “No”

For Yes:

( ) There must be only “Yes”.

**9.3. If the paper contains ONLY NON-clinical trials (*in vivo*) consider:**

**For Non-clinical trials:**

( ) Yes

( ) No

( ) Instead of assessing the RoB, the review authors must have used a satisfactory technique for assessing the limitations in individual studies that were included in the review.

**10. Did the review authors report on the sources of funding for the studies included in the review?**

For Yes:

( ) Must have reported on the sources of funding for individual studies included in the review.

( ) Yes

( ) No

Note: Reporting that the reviewers looked for this information, but it was not reported by study authors also qualifies.

**11. If meta-analysis was justified did the review authors use appropriate methods for statistical combination of results?**

**11.1 If the paper contains ONLY clinical studies, consider original evaluation:**

**RCTs**

For Yes:

( ) Yes

( ) No

( ) No meta-analysis conducted

( ) The authors justified combining the data in a meta-analysis.

( ) AND they used an appropriate weighted technique to combine study results and adjusted for heterogeneity if present.

( ) AND investigated the causes of any heterogeneity

**NRSI**

For Yes:

( ) The authors justified combining the data in a meta-analysis.

( ) Yes

( ) No

( ) No meta-analysis conducted

( ) AND they used an appropriate weighted technique to combine study results and adjusted for heterogeneity if present.

( ) AND they statistically combined effect estimates from NRSI that were adjusted for confounding, rather than combining raw data, or justified combining raw data when adjusted effect estimates were not available.

( ) AND they reported separate summary estimates for RCTs and NRSI separately when both were included in the review.

**11.2. If the paper contains clinical and NON-clinical trials (*in vivo*), consider:**

**For clinical trials:**

**RCTs**

For Yes:

( ) Yes

( ) No

( ) No meta-analysis conducted

( ) The authors justified combining the data in a meta-analysis.

( ) AND they used an appropriate weighted technique to combine study results and adjusted for heterogeneity if present.

( ) AND investigated the causes of any heterogeneity

**NRSI**

For Yes:

( ) The authors justified combining the data in a meta-analysis.

( ) Yes

( ) No

( ) No meta-analysis conducted

( ) AND they used an appropriate weighted technique to combine study results and adjusted for heterogeneity if present.

( ) AND they statistically combined effect estimates from NRSI that were adjusted for confounding, rather than combining raw data, or justified combining raw data when adjusted effect estimates were not available.

( ) AND they reported separate summary estimates for RCTs and NRSI separately when both were included in the review.

**For Non-clinical trials:**

( ) Yes

( ) No

For Yes:

( ) Yes

( ) No

( ) No meta-analysis conducted

( ) The authors justified combining the data in a meta-analysis.

( ) AND they used an appropriate weighted technique to combine study results and adjusted for heterogeneity if present.

( ) AND investigated the causes of any heterogeneity

( ) Yes

( ) Partial Yes

( ) No

**Total Result:**

For Partial Yes:

( ) If there is at least one “No”

For Yes:

( ) There must be only “Yes”.

**11.3. If the paper contains ONLY NON-clinical trials (*in vivo*) consider:**

**For Non-clinical trials:**

For Yes:

( ) Yes

( ) No

( ) No meta-analysis conducted

( ) The authors justified combining the data in a meta-analysis.

( ) AND they used an appropriate weighted technique to combine study results and adjusted for heterogeneity if present.

( ) AND investigated the causes of any heterogeneity

**12. If meta-analysis was performed did the review authors assess the potential impact of RoB in individual studies on the results of the meta-analysis or other evidence synthesis?**

**12.1 If the paper contains ONLY clinical studies, consider original evaluation:**

For Yes:

( ) Yes

( ) No

( ) No meta-analysis conducted

( ) included only low risk of bias RCTs.

( ) OR, if the pooled estimate was based on RCTs and/or NRSI at variable RoB, the authors performed analyses to investigate possible impact of RoB on summary estimates of effect.

**12.2. If the paper contains clinical and NON-clinical trials (*in vivo*), consider:**

**For clinical trials:**

For Yes:

( ) Yes

( ) No

( ) No meta-analysis conducted

( ) included only low risk of bias RCTs.

( ) OR, if the pooled estimate was based on RCTs and/or NRSI at variable RoB, the authors performed analyses to investigate possible impact of RoB on summary estimates of effect.

**For Non-clinical trials:** “RoB” became “limitations”

( ) Yes

( ) No

For Yes:

( ) Yes

( ) No

( ) No meta-analysis conducted

( ) Instead of accounting for RoB in individual studies, the review authors performed analyses to investigate possible impact of limitations on summary estimates of effect..

**Total Result:**

( ) Yes

( ) Partial Yes

( ) No

For Partial Yes:

( ) If there is at least one “No”

For Yes:

( ) There must be only “Yes”.

**12.3. If the paper contains ONLY NON-clinical trials (*in vivo*) consider:**

**For Non-clinical trials:** “RoB” became “limitations”.

( ) Yes

( ) No

( ) No meta-analysis conducted

For Yes:

( ) Instead of accounting for RoB in individual studies, the review authors performed analyses to investigate possible impact of limitations on summary estimates of effect..

**13. Did the review authors account for RoB in individual studies when interpreting/ discussing the results of the review?**

**13.1 If the paper contains ONLY clinical studies, consider original evaluation:**

For Yes:

( ) Yes

( ) No

( ) included only low risk of bias RCTs.

( ) OR, if RCTs with moderate or high RoB, or NRSI were included the review provided a discussion of the likely impact of RoB on the results.

**13.2. If the paper contains clinical and NON-clinical trials (in vivo), consider:**

**For clinical trials:**

For Yes:

( ) Yes

( ) No

( ) included only low risk of bias RCTs.

( ) OR, if RCTs with moderate or high RoB, or NRSI were included the review provided a discussion of the likely impact of RoB on the results.

**For Non-clinical trials:**

( ) Yes

( ) No

For Yes:

( ) Yes

( ) No

( ) Instead of accounting for RoB in individual studies, the review authors must have accounted for limitations when interpreting/ discussing the results of the review..

**Total Result:**

( ) Yes

( ) Partial Yes

( ) No

For Partial Yes:

( ) If there is at least one “No”

For Yes:

( ) There must be only “Yes”.

**13.3. If the paper contains ONLY NON-clinical trials (*in vivo*) consider:**

**For Non-clinical trials:** “RoB” became “limitations”.

( ) Yes

( ) No

For Yes:

( ) Instead of accounting for RoB in individual studies, the review authors must have accounted for limitations when interpreting/ discussing the results of the review.

**14. Did the review authors provide a satisfactory explanation for, and discussion of, any heterogeneity observed in the results of the review? Heterogeneity = inconsistency**

For Yes:

( ) There was no significant heterogeneity in the results.

( ) Yes

( ) No

( ) OR if heterogeneity was present the authors performed an investigation of sources of any heterogeneity in the results and discussed the impact of this on the results of the review.

**15. If they performed quantitative synthesis did the review authors carry out an adequate investigation of publication bias (small study bias) and discuss its likely impact on the results of the review?**

( ) Yes

( ) No

( ) No meta-analysis conducted

For Yes:

( ) performed graphical or statistical tests for publication bias and discussed the likelihood and magnitude of impact of publication bias.

**16. Did the review authors report any potential sources of conflict of interest, including any funding they received for conducting the review?**

For Yes:

( ) The authors reported no competing interests OR.

( ) Yes

( ) No

( ) The authors described their funding sources and how they managed potential conflicts of interest.
