# Supplementary material for: Effect of Moringa oleifera on inflammatory diseases: an umbrella review of 26 systematic reviews
Source: Front Pharmacol. 2025 May 19;16:1572337. doi: 10.3389/fphar.2025.1572337 (PMC12127422; doi:10.3389/fphar.2025.1572337)
Supplement: Supplementary file 3 [file Table3.docx]

| **Supplementary data 3:** A) General characteristics of included SRs.  Studies | Journal and Impact factor | Registration Number | Meta-analysis | Databases  Source | Search Time | Countries  Research group | Countries  Primary studies | Condition | Types of studies | Nº. of primary  studies | Quality Assessment |
| --- | --- | --- | --- | --- | --- | --- | --- | --- | --- | --- | --- |
| Popoola JO 2020 | Journal of Herbmed Pharmacology  0.356 | NR | N | PubMed, ScienceDirect, Web of Science, Scopus and Mendeley | 29 years (1990 - 2019) | Nigeria | India, Nigeria, South Africa, Italy, Bangladesh, Egypt, Cameroon, Malaysia, Iran, Brazil | Diabetes mellitus, Hypertension, Asthma, Cancer, Obesity, Arthritis | *In vitro, In vivo* (rats, mice) and clinical trial | Total of 63 studies. 7 studies involving activities: anti-inflammatory and antioxidant (2), cardioprotective (1), anticancer (3), wound healing(1)) and 9 for toxicity and safety evaluation | Procedure of Siqueira-Lima et al., 2017 |
| Kasali FM 2021 | Frontiers in Pharmacology  5.988 | NR | N | Google Scholar, Medline, PubMed, Scopus, ScienceDirect, the Wiley Online Library, Web of Science | 2005 - September 2021 | Democratic Republic of Congo, Uganda | Democratic Republic of Congo | Diabetes mellitus | Clinical trial | 34 studies of all plants. Only 3 about MO: ethnopharmacologica, 2 ethnobotanicals,  1 clinical trial | CAMARADES Jadad scale |
| Egbuna C  2021 | Current Topics in Medicinal Chemistry  3.570 | NR | N | PubMed, ScienceDirect, Google Scholar | January 2010 - October 2020 | Nigeria | NR | Diabetes mellitus | *In silico* | 84 studies (all compounds). One (1) In silico study about MO | NR |
| Phimarn W 2021 | Pharmacognosy Magazine  0.948 | NR | Y | PubMed, ScienceDirect, Scopus, and Thai Library Integrated System | from inception - December 2019 | Thailand | India, Thailand, Philippines | Diabetes mellitus and dyslipidemia. | Randomized controlled trials (RCTs) and non-RCTs | 7 clinical trials | Cochrane risk-of-bias tool and Jadad scale |
| Krawczyk M 2022 | Current Issues in Molecular Biology  2.976 | NR | Y | PubMed, MEDLINE, the Cochrane Library and Google Scholar | after the year 2000 | Poland | Table with "area of occurrence" indication: Africa and India | Diabetes mellitus | *In vivo* (rats) | 23 studies, 7 were about MO | NR |
|  |  |  |  |  |  |  |  |  |  |  |  |
| Studies | Journal and Impact factor | Registration Number | Meta-analysis | Databases  Source | Search Time | Countries  Research group | Countries  Primary studies | Condition | Types of studies | Nº. of primary  studies | Quality Assessment |
| Triantafillidis JK 2022 | Nutrients  6.706 | NR | N | PubMed, Web of Science, and Google Scholar | January 1993 - June 2021 | Greece | India. | Pancreatic Cancer | *In-vitro*, *In vivo* (mice) | 183 of all plants. 2 studies about MO (1 *in vitro* + 1 *in vivo*) | NR |
| Khazaei H 2020 | Phytotherapy Research  6.388 | NR | N | PubMed, Scopus, and Cochrane library | 2004 - August 2020 | Italy/Iran | NR | Diabetes mellitus associated neurodegenerative diseases | *In vivo* (rats) | 58 of all plants. 1 *in vivo* studie about MO | NR |
| Louisa M  2022 | Frontiers in Pharmacology  5.988 | NR | N | PubMed, Scopus, Google Scholar, and Cochrane Central Register of Controlled Trials | from inception - 1st of August 2021 | Indonesia | NR | Diabetes mellitus, obesity, hyperglycemia, dyslipidemia, hypertension. | RCTs and non-RCTs, *In vitro* and *In vivo* (animals) | 102 nonclinical studies and 7 clinical trials | NR |
| Watanabe S 2021 | Molecules  4.927 | NR | Y | PubMed and Embase | 2003 - 30 April 2021 | Japan | NR | Diabetes mellitus | *In vivo* (rodents) | 46 | Cochrane Collaboration “Risk of Bias” Tool |
| Karimi I  2022 | Asian Pac J Cancer Biol  1.892 | NR | N | PubMed | 1998 - 10 November 2021 | Iran | NR | Human colorectal cancer, leukemia, non-small cell, adenocarcinoma, breast cancer | *In vitro*, I*n vivo* chemically induced model, and tumor graft models | 71 | NR |
| Ramamurthy S 2021 | The Journal of Contemporary Dental Practice  0.902 | NR | N | PubMed/  Medline, the Cochrane Study register, Embase | 2000–2020 | India, Saudi Arabia | South Africa, Korea, USA, Malaysia, Italy, Brazil, Japan, Thailand, Kenya, India | Cancer, Inflammation | *In vitro* (cell lines of human or animal sources, both normal and tumor types) | 18 studies. 10 = Anticancer, 6=Antioxidant and antiinflammatory, 1=Wound healing, 1=Neurodifferentiatio, Adipogenesis, Osteogenesis | Risk of bias: Cochrane tool; Qualitatite synthesis: guidelines (Thomas and Harden and Bearman and Dawson) |
| Studies | Journal and Impact factor | Registration Number | Meta-analysis | Databases  Source | Search Time | Countries  Research group | Countries  Primary studies | Condition | Types of studies | Nº. of primary  studies | Quality Assessment |
| Redha AA 2021 | Journal of Functional Foods  5.223 | NR | N | PubMed and Scopus, and Google Scholar | 1st January 2010 - 6th June 2021 | Bahrain, United Kingdom | NR | Obesity | *In vitro* and *In vivo* studies (animal and human) | 36 studies: 8 *in vitro*, 24 *in vivo*, 1 involved *in vitro* and *in vivo*, 2 involved *in vivo* and clinical studies, and 1 study based on clinical trial only. | NR |
| Nova E  2020 | Nutrients  6.706 | NR | N | PubMed database | August 2019 - April 2020 | Spain | NR | Diabetes mellitus | *In vivo* | 33 animal studies and 8 human studies | NR |
| Nurul M  2020 | European Journal of Molecular & Clinical Medicine  0.7 | NR | N | Google Search and Pubmed | 2011 - 2020 | NR | NR | Anti-inflammatory activity in the human oral cavity (gingivitis and periodontitis | *In vitro*, *In vivo* (rats) | 10 studies with Anti-inflammatory activity | NR |
| Aumeeruddy MZ 2021 | Journal of Herbal Medicine  2.542 | NR | N | Sciencedirect, Medline/PubMed, and Google Scholar | 1988 - 2020 | Mauritius, Viet Nam | Bangladesh, Mexico, Burkina Faso | Obesity | Field ethnomedicinal studies | 108 of all plants.3 about MO (only referenced, without further information about the studies) | NR |
| Aumeeruddy MZ 2020 | South African Journal of Botany  3.111 | NR | N | Sciencedirect, Medline/PubMed, and Google Scholar | 1970 - 2019 | Mauritius, Viet Nam | Bangladesh, Benin, Eritrea, Ghana, India, Mauritius, Myanmar, Nigeria, Pakistan, Philippines, Reunion, Rodrigues, Sierra Leone, Thailand, Togo | Hypertension | Ethnobotanical field studies | 433 of all plants. 22 about MO (only referenced, without further information about the studies). | NR |
| Studies | Journal and Impact factor | Registration Number | Meta-analysis | Databases  Source | Search Time | Countries  Research group | Countries  Primary studies | Condition | Types of studies | Nº. of primary  studies | Quality Assessment |
| Da Silva MG 2022 | Evidence-Based Complementary and Alternative Medicine  2.064 | NR | N | Capes Periodical Portal, SciELO and BVS Portal | 2001 - 2020 | Brazil | NR | Hypertension | *In vivo* (rats), *in vitro* and *in silico* | 95 of all plants. 3 about MO (*in vivo,* *in vitro* and *in silico*) | NR |
| Nurhayati T 2024 | Journal of Nutrition and Metabolism  2.3 | NR | N | PubMed, Cochrane Library, Google Scholar, Scopus, and ScienceDirect | 2014 - 2023 | Indonesia | Indonesia, Mexico, Egypt, Nigeria, Iran, Saudi Arabia, South Korea, Pakistan, and Libya | Liver disease (non-alcoholic fatty liver disease) | *In vivo* (rat and mice) | 13 *in vivo* studies (8 rats e 5 mice) | Joanna Briggs Institute (JBI) Critical Appraisal Checklist |
| Hasim 2023 | Food Research  1.06 | NR | Y | Proquest, Science Direct, Ebsco, Cengage Library, and Emerald | Up to May 2021 | Indonesia | Indonesia | Obesity | *In vitro* | 5 of all plants. 1 about MO. | NR |
| Setyani W 2023 | Journal of Advanced Pharmaceutical Technology & Research  1.4 | NR | N | Scopus, PubMed, and DOAJ | 2011 – 2021 | Indonesia | NR | Diabetes mellitus | *In vitro, in vivo, and in silico* | 32 studies: 16 *in vivo*, 10 *in vitro*, 6 *in silico*. | NR |
| Salhab H 2023 | Journal of Health and Translational Medicine  0.2 | NR | N | Google Scholar, Science Direct, PubMed, and Scopus | 2009 - 2019 | Malaysia | Malaysia, India, Italy, South Korea, South Africa, Saudi Arabia, Thailand, Indochina, Egypt, France, and the United States | Cancer | *In vitro* and *in vivo* | 44 studies: 37 *in vitro*, 3 *in vivo* and 4 of both designs | NR |
| Studies | Journal and Impact factor | Registration Number | Meta-analysis | Databases  Source | Search Time | Countries  Research group | Countries  Primary studies | Condition | Types of studies | Nº. of primary  studies | Quality Assessment |
| Das M 2023 | Journal of Herbmed Pharmacology  1.32 | NR | Y | Web of Science, Scopus, PubMed, and PubMed Central | from the year 2015 | India | NR | Oral diseases (gingivitis, periodontitis, oral inflammation, oral cancer) | *In vivo* (patients and animal subjects) and *in vitro* | 9 studies: 4 *in vivo* (animals) and 2 *in vivo* (humans) | NR |
| Hasim H 2023 | Indonesian Journal of Chemistry  1.34 | NR | Y | Scopus, Science Direct, Proquest, Cengage Library, Ebsco, and Emerald | 2011 - 2021 | Indonesia | Egypt, the UK, South Africa, India, Thailand, and Jordan | Obesity | *In vitro* | 19 studies: 1 about MO | NR |
| Sivanesan R K 2022 | Sains Malaysiana  0.7 | NR | N | Medline via Ovid Medline and Scopus | NR | Malaysia | NR | Obesity and dyslipidemia | *In vitro* and *in vivo* | 29 about MO: 21 reported on rats, 3 with mice and 1 each with rabbit, dairy cow and human | NR |
| Dubey S 2023 | International Journal of Pharmaceutical Sciences and Research  4.7 | NR | Y | Scopus, PubMed, Science Direct, Cochrane Library, Medicine, and Google Scholar | 2010 to June 2022 | India | NR | Wound healing | *In vitro* | 24 studies: 1 about MO | Y |
| Pasupuleti M K 2023 | Journal of International Society of Preventive and Community Dentistry  1.4 | NR | N | PubMed, Scopus, and Web of Science | 2010 - 2020 | India | NR | Cronic gingivitis | *In vivo* | 14 studies: 1 about MO | Cochrane |
|  |  |  |  |  |  |  |  |  |  |  |  |

| **Supplementary data 3:** B) Characteristics of the experimental models included in the SRs. |  |  |  | |  | |  | |  |  |  | |  | |  |  |
| --- | --- | --- | --- | --- | --- | --- | --- | --- | --- | --- | --- | --- | --- | --- | --- | --- |
| Studies | Used part plant | Forms | | Methods of extracts/  Preparation | | Solvent | | Design of experiment | | | | Adverse effects | | Key Finding | | |
| Popoola JO 2020 | Leaf, stem back, seed, root | Raw, extract, isolated compounds | | Powder. Extraction method: NR | | Water, Alcohol, Methanol | | Population: Cells: Human neuroblastoma, human esophageal cells cancer, PC-3; Male Wistar rats, Sprague-Dawley rats, mice; Male and female humans. Route of administration: Animals: I.P, P.O. Humans: P.O. Dosage range: Animals: 1000mg/kg – 6400mg/kg extract. Duration range: Animals: 2 weeks – 2 months | | | | No adverse effects based on histopathology tests, relative organ weights, and toxicity biomarkers | | MO (leaves, stem and bark or whole leaves) could be safe for consumption as a vegetable, as well as a therapeutic agent to fight diseases and possibly enhance healthy living. This SR concludes that MO could be further explored in the search for cheaper and safer therapeutic agents against diseases. | | |
| Kasali FM 2021 | Leaf, flower | Extract, raw | | Infusion, decoction,  trituration. Clinical trial: powder | | NR | | NR | | | | NR | | MO is relatively non-toxic in animal models (LD50= 1585mg/kg bw > 500 mg/kg). The clinical study analyzed was considered of poor quality Jadad = 2 (Randomization = 0, Blinding = 2 Withdrawals and dropouts = 0). | | |
| Egbuna C  2021 | NR | NR | | NR | | NR | | NR | | | | NR | | Effective binding within the active binding pocket of the targeted protein is highly selective to their targeted protein and docked strongly to the mutated diabetic protein. | | |
| Phimarn W 2021 | Leaf | Raw | | Powder, capsules, tablets | | NR | | Population: 257 participants (89 had DM-2 and 108 had dyslipidemia), mean age ranged from 18 to 60 years. Dosage range: 1.5 – 8g/day. Duration range: follow‑up duration was 28–90 days | | | | Frequent urination, headache, cough, and urine color change (mentioned only two studies recruited). | | ↓ Blood glucose levels and ↑ HDL level. Administration of MO for >30 days could significantly ↓ FBS.  Limitations: (1) small number of trials and participants; (2) variations in product formulations, standardized methods and dose regimens (3) Only one study used a MO product standardized and reported the quantity of active compound (4) short study duration. | | |
| Krawczyk M 2022 | NR | Extract | | NR | | NR | | Population: ≈ 360 Rats (Albino, Wistar, Charles Foster strain), male and female, adults (8-12 weeks), weight 130 - 230g. | | | | NR | | ↓ glycemia: inconsistent results. Insulin level: the results were not precise because of the differences among the subgroups; ↓ SOD activity = ↓ oxidative stress; CAT activity: some discrepancies in the results MO extract reveals antiperoxidative properties to protect β cells against ROS. | | |
| Studies | Used part plant | Forms | | Methods of extracts/  Preparation | | Solvent | | Design of experiment | | | | Adverse effects | | Key Finding | | |
| Triantafillidis JK 2022 | *In vitro*: leaf *In vivo*: NR | *In vitro*: Extract  *In vivo*: NR | | NR | | *In vitro*: water  *In vivo*: NR | | Population: Pancreatic human cells. Nude mice. | | | | Some studies suggest that it may cause adverse effects when consumed in large quantities. It may interfere with prescription drugs affecting cytochrome P450. | | MO administration combined with radiation therapy has an additional inhibitory effect by overcoming the radioresistance of PC cells. The supplementation with MO leaf extract is potentially toxic at levels > 3000 mg/kg of BW, being safe at levels < 1000 mg/kg. MO aqueous leaf extract inhibits of NF-ĸB signaling pathway, and increases of the efficacy of chemotherapy with cisplatin in human PCcells. | | |
| Khazaei H 2020 | Leaves | NR | | NR | | NR | | Population: Rats. Route of administration: P.O (Supplementary diet) | | | | NR | | Quercetin and Gallic acid are among the main compounds involved in the therapeutic effect against the neurodegenerative complications caused by DM. Quercetin: considered the most promising active, being able to act as an adjunct therapy for hyperglycemia and its complications (especially cognitive declines). | | |
| Louisa M  2022 | Leaves, seeds, pods, flowers, root | Extract, fractionated extract, raw, isolated compounds, fractions, oil, nanoparticles | | Powder, capsules, Cold pressing, maceration, decoction, infusion, soxlet, lyophilization, hydrodestilation, ultrasonic | | Water, ethanol, methanol, ethyl acetate fractions, petroleum ether. | | Population: Cells: Raw 264.7, HepG2, RBL-2H3, HaCaT, J774A.1, 3T3-L1 adipocytes, LO2; Rats wistar and swiss albino, db/db mice, new zealand rabbits (males and females); 10 to 68 subjects (sex NR). Route of administration: Animals: P.O., I.P; Humans: P.O. (capsule or add to a meal). Dosage range: *In vitro*: 10 ng/mL - 1000 ug/mL extract; Animals: 200–800 mg/kg; Humans: 32–128 mg/60 kg (powder or extract). Duration range: Animals: 1 to 24 weeks; Humans: 1 day to 10 weeks. | | | | Clinical trials: no notable increase in adverse events when given up to 4 g. A study using a higher dose (40–60 g) caused changes in routine hematology parameters and increased cholesterol levels. | | Several animal models provided robust beneficial effects for the use of MO in the treatment of CMD. *In vitro* and animal toxicity studies demonstrated high safety. Animal studies: relatively high dose of LD50, ranging from 2,000 to 5,000 mg/kgBW. Long-term clinical trials applying standardized preparations and doses of MO extracts, particularly in T2DM and obese individuals, are needed to confirm efficacy and safety in patients. | | |
| Watanabe S 2021 | Leaves, seed, pods, fruits, aerial parts, stem bark, flower | NR | | NR | | NR | | Population: 10 to 40 (Mice, db/db mice or rats) in each study, males and females, weight 74,6g to 500g, age 2 weeks to 12 months. Route of administration: P.O (gavage). Duration range: 1 to 24 weeks. | | | | NR | | MO reduces blood glucose, serum/plasma TG, and TC levels of DM rodent models. MO effects on blood glucose levels differ significantly among DM models: reduction in chemical-, diet-induced and db/db mice, but not in dexa-induced. Significant evidence of publication bias in chemical-induced models and of heterogeneity in MA. | | |
| Studies | Used part plant | Forms | | Methods of extracts/  Preparation | | Solvent | | Design of experiment | | | | Adverse effects | | Key Finding | | |
| Karimi I  2022 | leaves, seeds, pods, phytocompounds | Extract, fractionated extract, oil, isolated compounds, | | Maceration, decoction, percolation, cold pressing, powder, lyophilization | | Water, ethanol, methanol, hydro-alcohol; dichloromethane; n-hexane, ethyl acetate, chloroform | | Population: MCF-7, HepG2, and Hela cells; Animals: Balb/c mouse, nude mice, Sprague-Dawley rats, CD1 mice (males and females). | | | | NR | | Phenolic compounds probably are involved in the cytotoxicity triggered by MO. Is not any report in terms of antitumor activity of MO against metastatic cancers. Leaves of MO as a major botanical part have been used for its anticancer activities. Moringin has been reported as the main component of seeds and pods, and it and its analogues are considered as putative phyto-oncolytics or phyto-oncostatics. Human colorectal cancer was the main tumor phenotype studied. The antitumor effects of MO were mainly due to the induction of apoptosis, anti-proliferation, anti-angiogenesis, and DNA/RNA fragmentation. | | |
| Ramamurthy S 2021 | Leaf, seed, flower, pod, bark, root | Extract, fractionated extract, microvesicle, isolated compounds | | Hot treated/boiled, cold treated/frozen , lyophilization, microvesicle extraction, soxlet | | Water,ethanol, butanol, ethyl acetate, chloroform,  hexane | | Population: Normal cell lines: monkey kidney cells, human periodontal stem cells, murine macrophage cell lines, and human dermal fibroblasts. Cancer cell lines: lung cancer cells, breast cancer, colorectal cancer, pulmonary mucoepidermoid carcinoma, larynx carcinoma, small cell lung cancer, and hepatocellular carcinoma. | | | | NR | | MO leaf extracts achieved the most potent beneficial effects, while MO seed extracts were the weakest. MO leaves were the best sources of extracts, especially under aqueous and alcoholic extraction methods. Heat treatment seemed to lower the effect; cold treatment seems to be preferred. Fractionation has been reported to generate higher potency, but the adverse effects were also magnified. MO extracts had significant anti-inflammatory, antiproliferative, and antioxidant properties. The antioxidant activity was seen to be upregulated and anti-inflammatory activity was downregulated, both activities seemed to correlate with the flavonoid content of the extracts. MO had cytotoxic effect in cancer cell lines (antiproliferation and apoptotic activities), and protective effect in normal cell lines. The antioxidant properties were due to the suppression of MAD and an increase in ascorbic acid, SOD, and GPx. The NFκB pathway seems to be the major point of MO activity. | | |
| Studies | Used part plant | Forms | | Methods of extracts/  Preparation | | Solvent | | Design of experiment | | | | Adverse effects | | Key Finding | | |
| Redha AA 2021 | Leaf, seed, aerial parts of plant | Extract, raw, oil, isolated compounds | | Powder, hard gelatine capsule | | Methanol, ethanol, petroleum ether, Water, Oil, Hydroalcoolic | | Population: Cells/Enzimes: 3T3-L1, Pancreatic lipase, cholesterol esterase, Human adipose tissue mesenchymal stem cells. Animals: Swiss albino, Albino wistar rat, Long Evans rat, Sprague dawley rat, C57BL/6J, C57BL/6 mice, C7BI6 mice, C57B1/6J mice, DDY mice; males and females, weight 120 - 200 g, age 3 -16 weeks; Humans: males and females, 44 - 55 years old, BMI 29-34 kg/m^2^. Route of administration: P.O. Dosage range: Animals: 125 - 800 mg/kg extracts; Humans: 20g MO leaves powder, 50g MO powder formula, 400mg extract. Duration range: Animals: 30 - 119 days; Humans: 20 - 56 days. | | | | NR | | MO leaves powder lowered the increment of the postprandial blood glucose in diabetic participants. The impact of MO on SOD and CAT is not clear as different studies have reported different findings, this can be associated with the dosage of extract and duration of the test. MO has shown clear anti-obesity potential in laboratory and animal studies, but in clinical trials the impact of MO has limited on body mass index, TC, LDL, and postprandial blood glucose. No clinical studies have been conducted to explore the regulation of adipogenesis genes in humans. | | |
| Nova E 2020 | Leaves, seeds, pods, stem bark | Extract, raw, isolated compounds | | cookies, powder (leaf, seed), capsules (leaf), tablet (leaf) | | Water, ethanol, methanol, n-hexane | | Population: Animals: Sprague dawley, wistar, Goto-kakizakis, Long evan, and Charles Foster albino Rats; C57BL/6, Albino, Swiss, C57BL/6J, C57B1/6J, db/db, and Swiss webster mices (adult, males and females); Humans: 73 men + 113 women + 60 gender unspecified, 20 - 71 years, 18,1 - 35 kg/m^2^. Route of administration: Animals: P.O (diet, gavage), I.P; Humans: P.O. Dosage range: Animals: Powder: 50 - 200 mg/kg; Aq: 100 - 300 mg/kg; Me: 150 - 600 mg/kg; Et: 150 - 500 mg/kg; N-hexane: 40 - 80 mg/Kg. Typical dose of the extracts ≈ 200–300 mg/kg. Humans 1 - 20g powder/day. Duration range: Animals: 1 day (single dose) - 24 weeks; Humans: 2 single occasions - 90 days. | | | | No adverse effects have been observed with a methanol extract up to a dose of 3000 mg/kg in rodent’s experiments. NR for human experiments. | | Animal studies: Significant improvements in blood glucose, both in fasted state and in response to a glucose tolerance test. The high fiber content in the MO leaves, the inhibition of intestinal glucose uptake and the improved glucose uptake in muscle and liver are associated with glycemic control; Most long-term studies with MeMo or EtMO found positive results on fasting glucose and OGTT; Lack of consistency in the effect on insulin levels and in the effect of MeMO or AqMO on FBG (normal rats).  Clinical trials: Few published studies with very variable designs and insufficient number of patients, making it difficult to reach a consensus on the indication of MO in treatment of DM.; Potential usefulness in postprandial studies; Cold water tea preparations are preferable to hot preparations. | | |
| Studies | Used part plant | Forms | | Methods of extracts/  Preparation | | Solvent | | Design of experiment | | | | Adverse effects | | Key Finding | | |
| Nurul M 2020 | Leaves, stem bark, seeds | Extracts | | NR | | Water, ethanol | | Population: Rats, mouse  Route of administration: P.O | | | | NR | | MO reduces inflammatory cytokines in the gingival tissue; decreases COX-2 expression; possess *in vitro* anti-inflammatory activity on LPS-stimulated macrophages by regulating cytokine and NO production; decreases leukocyte migration, myeloperoxidase activity and TNF-α and IL-1β levels | | |
| Aumeeruddy MZ 2021 | Fruit and root | Raw | | Decoction | | NR | | NR | | | | NR | | MO fruit taken a vegetable used for the management of obesity. | | |
| Aumeeruddy MZ 2020 | Leaf, bark, seed, stem, fruit, root, aerial parts | Extract, raw | | Decoction, infusion, maceration, juice, powder | | Water, alcohol | | NR | | | | NR | | All the MO plant is used – in the form of tea (infusion, decoction or maceration) or juice or eaten raw food/powdered – in the treatment of hypertension in Asia and Africa. | | |
| Da Silva MG 2022 | Leaves | NR | | NR | | NR | | Population: rats | | | | NR | | Alkaloids, such as reserpine and alstonine, reduce the availability of norepinephrine and, therefore, act as vasodilators. Flavonoids such as quercetin and rutin are primarily active in the myocardium and reduce cardiac output. Phenolic compounds are antioxidants responsible for scavenging free radicals, capable of minimizing the harmful effects of ROS, and considered potential for the prevention of cardiovascular diseases. | | |
| Nurhayati T 2024 | Leaf, seed, stem | Extract, fractionated extract, raw | | Powder | | Water, ethanol, methanol, ethyl acetate | | Population: rats and mice. Species: C57BL/6J mice, Wistar albino rat, Swiss albino mice. Sex: male. Dosage range: aqueous extract 62.5 mg/kg, ethanol extract 3.5 - 7 mg/kg, extract 150 – 800 mg/kg, fermented extract 250 mg/kg, ethyl acetate fraction 20.17 - 45.39 mg/kg, Methanolic extract 500 mg/kg. | | | | The aqueous leaf extract of MO did not result in any fatalities even at the highest administered dose of 5000 mg/kg or 6400 mg/kg body weight being considered safe for oral consumption, displaying no lethal effects during acute administration). | | The dosage of MO leaf extract of more than 200 mg/kg body weight showed more significant result of glucose and insulin profile, on lipid profile and MDA level, on liver histopathological feature and liver biomarkers than the lower dosage. Higher dosage of MO extract showed more reduction on the histopathological feature of the liver tissue, while lower dosage did not give any notable improvement on the histopathological feature of the liver tissue. | | |
| Studies | Used part plant | Forms | | Methods of extracts/  Preparation | | Solvent | | Design of experiment | | | | Adverse effects | | Key Finding | | |
| Hasim 2023 | NR | NR | | NR | | Methanol | | NR | | | | NR | | *Moringa oleifera* leaves demonstrated the most potential anti-obesity in comparison with other Indonesian medicinal plants. | | |
| Setyani W 2023 | Leaves | Extract, isolated compounds | | Direct maceration and successive maceration | | Water, ethanol, methanol, hexane, dichloromethane, ethyl acetate, n-butanol | | Population: Animals: Rats. C57BL, Wistar rat, albino rat, Sprague Dawley (male and female); age 6 weeks - 12 months; weight 100 - 250 g. Dosage range: Animals: aqueous extract 100 - 600 mg/kg, ethanolextract 100 - 400 mg/kg, methanolic extract 100 - 600 mg/kg. Route of administration: Animals: oral. Duration range: Animals: 1 week to 12 weeks. | | | | NR | | Quercetin exhibited the best affinity toward the SGLT-2 receptor. Myricitrin, quercetin, and polydatin bound to the main site while interacting with residues ARG442 and GLU411 at the catalytic site. Kaempferol had the highest affinity to the GLUT4 transporter. stevioside had an affinity to the of α-amylase and butyloxycarbonyl to the DPP-4, α-amylase, and α-glucosidase. | | |
| Salhab H 2023 | Leaves, fruits, roots, seeds, pods, stem, whole plant | Extract | | NR | | Water, methanol, ethanol and n-hexane | | Population: rats and mice. Immune deficient athymic CD-1 nude mice, mice (Mus musculus), Swiss albino mice and Wistar rats. Age: 3 - 8 weeks-old. Sex: male. Wight: rats 140 - 160 g and mice 15 - 27 g. Route of administration: orally and intraperitoneally. Dosage range: 10 mL/kg, 2 - 500 mg/kg, 0.5 - 1.5 mg/g. Duration range: 5 - 7 days and 6 - 20 weeks. | | | | NR | | MO is one of the most promising medicinal plants for preventing and treating cancer. One of the most widely used natural compounds against cancer is polyphenols found in plants. The most important anticancer mechanism of dietary polyphenols is the induction of apoptosis. | | |
| Das M 2023 | Leaves | Extract, crude extract | | NR | | NR | | Population: humans, rats and guinea pigs. Route of administration: Animals: intraorally by means of insulin sonde. Humans: lozenge, Mouthwash. Dosage range: Animals: 0.1 mL; 3.125 - 9.375%. Humans 145.86 mg, 0.02 %. Duration range: Animals: 1 - 30 days; Humans: 14 - 28 days. | | | | NR | | Due to limited data, the efficacy of MO extracts in the management of oral diseases on *in vitro* level cannot be confirmed. The Interpretation regarding in vivo studies was wrong, as in vivo efficacy could not be confirmed either. | | |
| Hasim H 2023 | NR | NR | | NR | | Methanol | | NR | | | | NR | | The activity of MO as an anti-obesity agent is associated with the presence of niazirin, which belongs to a member of phenolic glycoside. | | |
| Studies | Used part plant | Forms | | Methods of extracts/  Preparation | | Solvent | | Design of experiment | | | | Adverse effects | | Key Finding | | |
| Sivanesan R K 2022 | Leaves, whole plant and seed | Extract | | Powder | | Methanol, ethanol, water and hexane | | Population: Animals: Rats, mice, rabbits, dairy cows. Long Evans rats, Wistar rats, C57BL/6J mice, New Zealand white rabbits, Holstein cows, Swiss Albino mice, Sprague Dawley rats (4 -17 weeks, males and females). Humans (males and females). Route of administration: Animals and Humans: Orally. Dosage range: Animals: Powder leaves 0.5% - 30%; Methanolic extract 100 - 600 mg/kg; Ethanolic extract 100 - 600 mg/kg; Aqueous extract 200 - 800 mg/kg. Humans 0.03 g/kg, 0.07 g/kg MO powder. Duration range: Animals: 14 – 90 days; Humans: 14 days. | | | | NR | | The present review showed that different extract, explant, and dosages of MO are essential anti-obesity and antihyperlipidemic agents that lead to a reduction in weight loss and serum lipids. More human intervention should be focused on as we could clearly observe the lack of clinical evidence on the effects of MO on different human population. Considering that the results from the animal studies were promising, therefore, it can be recommended to patients that having complication with high lipid levels and obesity. | | |
| Dubey S 2023 | Seeds | Extract | | NR | | Hydroalcoholic | | Population: fibroin cell culture. | | | | NR | | Seeds of this plant have high efficiency of wound healing activity in both control and standard treatment.  In the case of the incision model, the application of the topical array has been done by enhancing the clarification of the breaking strength. | | |
| Pasupuleti M K 2023 | NR | Extract | | NR | | NR | | Population: rats. | | | | NR | | MO can be a promising alternative for managing chronic gingivitis. | | |
|  |  |  | |  | |  | |  | | | |  | |  | | |

RCT: randomized controlled trials; MO: *Moringa oleifera*; NR: Not reported; N: No; Y: Yes. MO: *Moringa oleifera*; NR: Not reported; LDL: low‑density lipoprotein; HDL: high‑density lipoprotein; TC: total cholesterol; TG: triglyceride; FBG: fasting blood glucose MDA: malondialdehyde; ROS: reactive oxygen species; SOD: superoxide dismutase; CAT: catalase; GPx: glutathione peroxidase; NF-κβ: nuclear factor- Kβ; DM: diabetes mellitus; OGTT: Oral glucose tolerance test; AqMO: aqueous extract of MO; EtMO: ethanolic extract of MO; MeMO: methanolic extract of MO; BW: body weight; CMD: cardiometabolic disorders; I.P: intraperitoneal; P.O: oral.
