# Supplementary material for: Effect of Moringa oleifera on inflammatory diseases: an umbrella review of 26 systematic reviews
Source: Front Pharmacol. 2025 May 19;16:1572337. doi: 10.3389/fphar.2025.1572337 (PMC12127422; doi:10.3389/fphar.2025.1572337)
Supplement: Supplementary file 4 [file Table4.docx]

**Supplementary data 4:** Preparation forms used in clinical trials (primary articles).

| Clinical trial | Preparation | Dosis |
| --- | --- | --- |
| Agrawal and Mehta, 2008 | MO seed kernels | 3g/day |
| Ahmad et al., 2017 | Cookies MO leaf powder | Cookies containing MO leaf powder (5% w/w) |
| Anthanont, 2016 | MO leaf powder capsules | 0, 1, 2 and 4 g |
| Baipakdee, 2013 | MO capsule (powder leaf) | 1500 mg/day |
| Dominguez-Rodrigues M et al., 2016 | MO leaves extract (method extraction NR) | NR |
| Ezzat et al., 2020 | MO leaves extract (maceration with 70% ethanol) | 400 mg/capsule |
| Fombang and Saa, 2016 | MO leaf tea | 200 or 400 mL |
| Giridhari et al., 2011 | MO leaf tablets | 2 Drumstick leaf powder (98.34%) tablets/day |
| Ifeoma, 2020 | Steamed MO leaves | 20, 40, 60 g |
| Kumar K and Mandapaka, 2013 | Dry MO leaves powder formula | 50g/day |
| Kumari, 2010 | MO leaf powder | 8 g/day |
| Kushwaha et al., 2014 | MO powder | 7 g/day |
| Leone et al., 2018 | MO leaf powder (dried and ground) | 20g |
| Nambiar et al., 2010 | Tablets | 575 mg 8 tablets/day |
| Sandoval and Jimeno, 2013 | MO capsule (powder leaf) | 2100 mg/day |
| Taweerutchana et al., 2017 | MO powdered leaf capsule | 8 g/day |
| Seriki et al., 2015 | MO powder (leaf) | 0.03 g/kg and 0.07 g/kg/day |
| Luetragoon et al., 2021 | Lozenge (MO leaf extract + C. cinereum over ground parts extract - maceration with 95% ethanol) | MO extract (145.86 mg/lozenge) and CC extract per (129.64 mg/lozenge), 3 lozenges per day |
| Buakaew et al., 2021 | Mouthwash (MO + Citrus hystrix leaf extracts maceration with 95% ethanol) | Mouthwash contained ethanolic extracts of MO + Citrus hystrix leaves (0.015% w/w), 10–15 mL twice daily for 30 s |
